# HMGCR‐Driven Cholesterol Metabolism Promotes Osteoarthritis Progression by Accelerating Synovial Fibroblast Senescence

**DOI:** 10.1002/advs.76498

**Published:** 2026-07-13

**Authors:** Xiaoqi Zhang, Siru Zhou, Xi Liu, Wei Xiang, Xiuqin Peng, Yu Tian, Song Li, Hailin Wang, Bo Liao, Shunzheng Fang, Runze Jin, Jinhui Wu, Chuanqing Bai, Wei Zhang, Bo Huang, Bingfei Li, Lifeng Chen, Lin Chen, Bin Zhang, Zhenhong Ni

**Affiliations:** ^1^ Department of Rehabilitation Medicine Army Medical Center Daping Hospital Army Medical University of PLA Chongqing China; ^2^ War Trauma Medical Center State Key Laboratory of Trauma and Chemical Poisoning Army Medical Center Daping Hospital Army Medical University Chongqing China; ^3^ Department of Otorhinolaryngology and Maxillofacial Surgery The 988th Hospital of the Joint Logistics Support Force Zhengzhou China; ^4^ Metabolism and Repair Laboratory For Prevention and Rehabilitation of Training Injuries State Key Laboratory of Trauma and Chemical Poisoning Trauma Center Research Institute of Surgery Army Medical Center Daping Hospital Army Medical University of PLA Chongqing China; ^5^ Department of Rehabilitation Medicine Key Laboratory of Physical Medicine and Precision Rehabilitation of Chongqing Municipal Health Commission The First Affiliated Hospital of Chongqing Medical University Chongqing China; ^6^ Department of Emergency Medicine Army Medical Center Daping Hospital Army Medical University of PLA Chongqing China; ^7^ Rehabilitation Center Key Specialty of Neck and Low Back Pain Rehabilitation Xingcheng Special Duty Sanatorium Xingcheng China

**Keywords:** cellular senescence, HMGCR, osteoarthritis, synovial fibroblast

## Abstract

Dysregulated cholesterol metabolism has been implicated in several aging‐associated disorders, including osteoarthritis (OA). 3‐Hydroxy‐3‐methylglutaryl‐CoA reductase (HMGCR) is the key enzyme in cholesterol biosynthesis, but the role of HMGCR in OA and the underlying mechanism remain unclear. In this study, we found that HMGCR^+^ fibroblasts (FLSs) were significantly increased in the knee synovium of OA patients and OA mice. Elevated expression of HMGCR in synovial FLSs was positively correlated with cellular senescence and OA progression. The inhibition of HMGCR reversed cellular senescence of FLSs induced by TNF‐α in vitro. Additionally, the HMGCR‐mediated cholesterol biosynthetic pathway contributed to cellular senescence. Targeted inhibition of HMGCR in synovial FLSs via intra‐articular adeno‐associated virus delivery effectively reversed cellular senescence of FLSs and mitigated synovitis, cartilage degradation, and pain behaviors in OA mice. Mechanistically, the phosphorylation of AKT1 at Ser473 enhanced its binding to Lys140 of Insig1, facilitating AKT1–Insig1 complex formation. The activation of AKT1 induced the phosphorylation of Insig1 at Ser189 and the dissociation of Insig1 from sterol regulatory element‑binding protein cleavage‑activating protein (SCAP), which contributed to HMGCR transcription and cellular senescence of FLSs. Collectively, our study reveals a novel mechanism of HMGCR‐driven cellular senescence of FLSs in OA, which highlights HMGCR as a promising therapeutic target for OA.

## Graphical abstract

In the pathological context of osteoarthritis (OA), the phosphorylation of AKT1 at Ser473 enhances its binding to Lys140 of Insig1, which facilitates the formation of AKT1–Insig1 complex. Subsequently, the activation of AKT1 promotes the phosphorylation of Insig1 at Ser189, potentially enhancing the dissociation of Insig1 from sterol regulatory element‑binding protein cleavage‑activating protein and the transcription of 3‐Hydroxy 3‐methylglutaryl‐CoA reductase, which ultimately contributes to cellular senescence in fibroblasts and promotes OA progression.

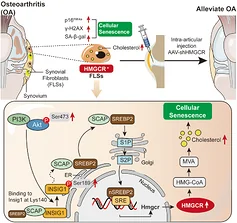

## 1 Introduction

Osteoarthritis (OA) is a highly prevalent degenerative disease, characterized by progressive cartilage degradation, subchondral bone sclerosis, and chronic synovitis [1, 2]. The synovium is a thin membrane that lines the surfaces of structures within the joint. The synovial tissue contains a variety of cell types, including fibroblasts (FLSs), macrophages, mast cells, adipocytes, and endothelial cells [3]. Synovitis serves as an independent etiological factor and a critical pathological hallmark of OA, which is closely correlated with clinical symptoms such as pain and joint swelling [4, 5]. A previous study reported that more than 89% of knee OA patients had synovitis [6]. Inhibiting synovitis is a potential strategy for delaying the progression of OA [7]. To date, the roles and molecular mechanisms of synovitis during OA progression remain elusive.

Cholesterol is an important lipid molecule that is synthesized by all mammalian cells. Cholesterol primarily localizes to cell membranes, where it associates with neighboring lipids to orchestrate the rigidity, fluidity, and permeability of the bilayer [8]. Meanwhile, cholesterol is a precursor to bile acids and steroid hormones and can regulate diverse signaling cascades during cellular proliferation, differentiation, and senescence [9, 10]. Dysregulated cholesterol metabolism has been linked to several aging‐associated disorders, including Alzheimer's disease, atherosclerosis, osteoporosis, immune dysfunction, and OA [11]. A previous study found that dysregulation of the cholesterol–metabolic axis, CH25H‐CYP7B1‐RORα, in chondrocytes accelerated OA‐related cartilage degradation and osteophyte formation [12]. Regulating serum cholesterol levels through cholesterol‐lowering drugs or controlling intracellular cholesterol levels in chondrocytes could alleviate OA progression [13]. However, the role of cholesterol metabolism in the OA synovium remains relatively underexplored.

3‑Hydroxy‑3‑methylglutaryl‑CoA reductase (HMGCR) serves as the rate‐limiting enzyme in the mevalonate (MVA) pathway, which is part of cholesterol biosynthesis and facilitates the endogenous biosynthesis of several polyisoprenoids, including ubiquinone, prenyl groups, and dolichol [14]. Aberrant regulation of HMGCR contributes to the pathogenesis of various diseases, including hepatocellular carcinoma, nonalcoholic steatohepatitis, and schnyder crystalline corneal dystrophy [15, 16, 17]. Recent studies found that HMGCR‐related pathway in chondrocytes and skeletal muscle cells played important roles in OA progression. Upregulated HMGCR in chondrocytes is crucially involved in OA pathogenesis [18]. Additionally, our group recently discovered that the mTORC1‐HMGCR pathway contributed to OA‐related muscle atrophy via a reduction in endogenous coenzyme Q10 (CoQ10) [19]. Nevertheless, the role of the HMGCR‐mediated cholesterol–metabolic axis in OA synovitis and the molecular pathways involved are still poorly understood.

In this study, we revealed the accumulation of HMGCR^+^ FLSs in the synovium of OA patients and the destabilization of the medial meniscus surgery (DMM)‐induced OA mice. Interestingly, these HMGCR^+^ FLSs exhibited a senescent phenotype. Furthermore, HMGCR‐mediated cholesterol metabolites contributed to cellular senescence of FLSs. Mechanistically, the activation of AKT1 in senescent FLSs promoted Insig1 phosphorylation through its interaction with AKT1, triggering its dissociation from sterol regulatory element‑binding protein cleavage‑activating protein (SCAP) and thereby enhancing HMGCR transcription. Upregulation of HMGCR resulted in sustained synthesis of cholesterol metabolites, which further accelerated cellular senescence. Inhibition of HMGCR in synovial fibroblasts alleviated cellular senescence, pain‐related behaviors, synovitis, and cartilage degradation in vivo, which offers a promising interventional approach for OA treatment.

## 2 Results

### 2.1 HMGCR^+^ Fibroblasts are Significantly Increased in the Synovium During the Progression of OA

We first evaluated the expression of HMGCR in the synovium of OA patients. Proteins were extracted from synovial tissues and subjected to western blotting (WB) analysis. Compared with the normal control group, HMGCR expression levels in synovial tissues from OA patients were significantly elevated (Figure 1A). Additionally, immunohistochemical staining showed that synovial tissues from OA patients exhibited both the increased thickness and the marked upregulation of HMGCR expression versus the normal control (Figure 1B). Compared with the Sham group, an elevated level of HMGCR protein was observed in the synovial tissue of DMM‐induced OA mice (Figure 1C). To determine the cell type highly expressing HMGCR in the synovium, we selected and integrated single‐cell RNA sequencing (scRNA‐seq) data from the synovium of an experimental OA mouse model (data from GSE211584) [20]. Based on the known marker genes, we categorized these cells into seven clusters, including FLSs, macrophages, endothelial cells, tenocytes, Schwann cells, pericytes, and lymphoendothelial cells (Figure 1D,E). Through comparative analysis of HMGCR^+^ cell proportions across various synovial cell types between the OA and control group, we observed that fibroblasts and pericytes exhibited higher expression of HMGCR than other cell types (Figure 1F). As FLS is a predominant cell type in the synovium, immunofluorescence double staining for HMGCR and PDGFR‐α (a FLS marker) in the synovium of OA patients and mice was performed. The results demonstrated that HMGCR^+^ FLSs were increased in the synovium of both OA patients and OA mice, compared to the respective normal groups (Figure 1G,H). Taken together, these results reveal that HMGCR^+^ FLSs are significantly increased in the OA synovium.

**FIGURE 1:**
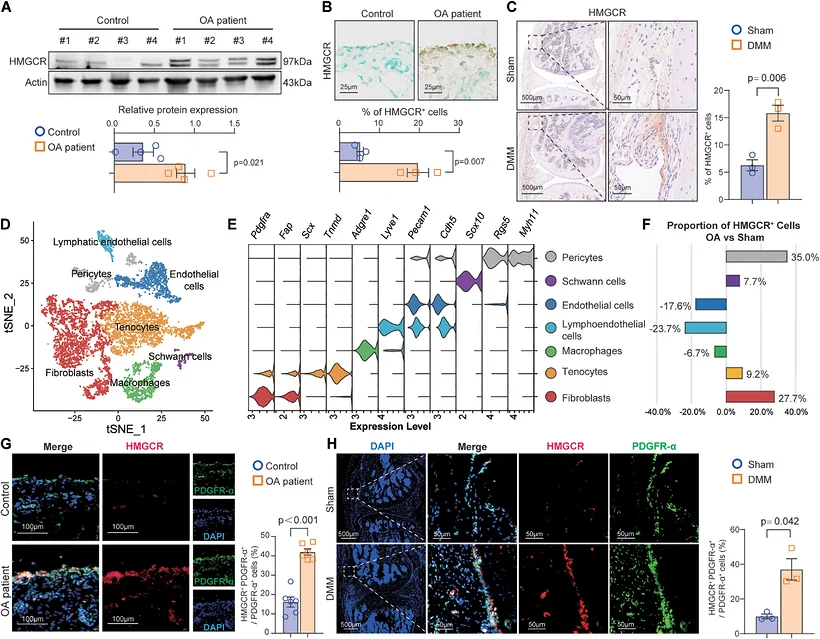
HMGCR^+^ synovial FLSs exhibit a significant increase during OA progression. (A) WB analysis of HMGCR protein levels in synovial tissues from non‐OA and OA patients. *n* = 4 per group. (B,C) Representative immunohistochemical staining images and quantification of HMGCR^+^ cells in synovial tissues from OA patients and DMM‐induced OA mice, compared with the normal control. *n* = 3 per group. (D) Cell clustering of cells from scRNA‐seq data in synovial tissues from OA and Sham mice (data from GSE211584) using UMAP. (E) Violin distributions of signature genes for synovial cell types in (D). (F) Comparative analysis of the proportions of HMGCR^+^ cells across synovial cell populations in OA mice relative to Sham mice. (G,H) Representative immunofluorescence staining images and quantification of the percentage of HMGCR^+^ PDGFR‐α^+^ cells in PDGFR‐α^+^ cells. Samples were obtained from OA patients (*n* = 6) and DMM‐induced OA mice (*n* = 3). All statistical tests were two‐sided. Student's *t*‐test (A–C, G). Welch's *t*‐test (H). WB, western blot; UMAP, uniform manifold approximation and projection.

### 2.2 Elevated HMGCR is Associated With Cellular Senescence of Synovial Fibroblasts in OA Progression

To further investigate the biological processes in HMGCR^+^ FLSs, we reanalyzed and classified the FLSs in experimental OA mice from the scRNA‐seq data described above into HMGCR^+^ or HMGCR^−^ FLSs. Enrichment of numerous signaling pathways was observed in HMGCR^+^ cells via the gene ontology enrichment analysis (GO Enrichment), and several pathways were related to cellular senescence (Figure 2A). Cellular senescence refers to the process by which cells gradually lose their normal functions and capacity to divide over time, which is characterized by hallmarks such as cell‐cycle arrest and the senescence‐associated secretory phenotype (SASP) [21]. Emerging evidence has confirmed that abundant senescent FLSs are present in the OA synovium and play a crucial role in synovitis and cartilage degradation during OA progression [22]. Furthermore, targeted inhibition of methyltransferase‐like 3 (METTL3) suppressed senescence of FLSs, thereby attenuating DMM‐induced cartilage destruction [23]. Moreover, GATD3A deficiency led to FLSs senescence via mitochondrial dysfunction, whereas intra‐articular delivery of recombinant adeno‐associated virus (AAV) encoding GATD3A markedly mitigated OA phenotypes in mice [24]. However, the detailed mechanisms underlying FLSs senescence in OA remain unclear. Of note, altered metabolic patterns are important hallmarks of senescent cells [25]. Recent evidence has revealed that cholesterol overload is closely related to cellular senescence [26, 27, 28, 29, 30]. The accumulation of cholesterol in lysosomes could drive cellular senescence, where the rerouted exporter ABCA1 facilitated the formation of cholesterol‐rich microdomains that activated mTORC1 and further reinforced SASP [26]. In particular, the cholesterol biosynthetic branch of the MVA pathway‐induced senescence via an ERRα‐mediated mitochondrial program [27]. It remains unknown whether HMGCR, as the rate‐limiting enzyme of the MVA pathway, is involved in FLSs senescence during OA progression.

**FIGURE 2:**
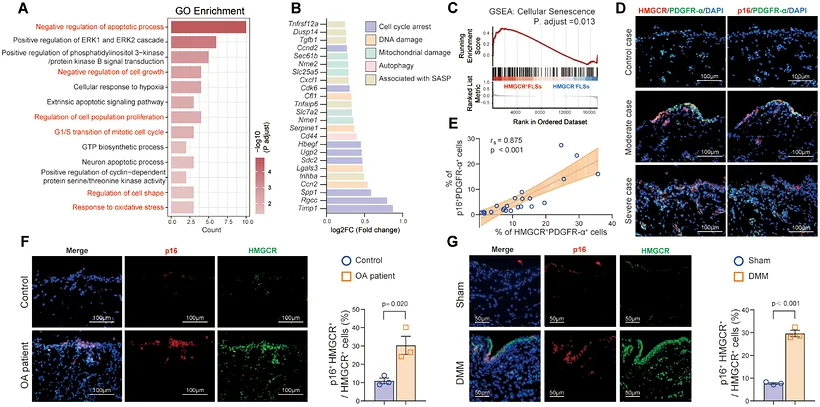
High expression of HMGCR is associated with cellular senescence in synovial fibroblasts during OA progression. (A) GO enrichment analysis showing enriched senescence‐associated terms (highlighted in red) in HMGCR^+^ FLSs compared to HMGCR^−^ FLSs, based on synovial data from experimental OA mice (data from GSE211584). (B) Senescence‐associated genes enriched in HMGCR^+^ FLSs from scRNA‐seq data in synovium of OA mice. (C) GSEA of ‘Cellular Senescence’ gene sets in HMGCR^+^ versus HMGCR^−^ FLSs. (D) Representative immunofluorescence staining images of HMGCR and p16 in PDGFR‐α^+^ FLSs of human synovium across varying inflammatory grades. Two adjacent sections from one sample were used separately to show the expression of HMGCR and p16. (E) Spearman's correlation analysis between percentages of HMGCR^+^ PDGFR‐α^+^ cells and p16^+^ PDGFR‐α^+^ cells in clinical synovial tissues. *n* = 23 samples. (F,G) Representative immunofluorescence staining and quantification of p16^+^ cells in HMGCR^+^ cells from synovial tissues in OA patients (*n* = 3) and OA mice (*n* = 3). All statistical tests were two‐sided. Student's *t*‐test (F,G).

To further investigate the function of HMGCR in FLSs senescence, we analyzed the differential expression of senescence‐associated genes between HMGCR^+^ and HMGCR^−^ FLSs from GSE211584 data. Key hallmarks of senescence, such as cell cycle arrest, DNA damage, mitochondrial dysfunction, impaired autophagy, and the SASP, were all prominently upregulated in HMGCR^+^ FLSs (Figure 2B). Furthermore, gene set enrichment analysis (GSEA) also showed that cellular senescence was significantly enriched in HMGCR^+^ FLSs (Figure 2C). In two adjacent sections, we performed double staining for HMGCR/ PDGFR‐α and p16/ PDGFR‐α, respectively (Figure 2D). Linear regression analysis revealed a positive correlation between frequencies of p16^+^ FLSs and HMGCR^+^ FLSs (Figure 2E). Additionally, we analyzed correlations between the proportion of HMGCR^+^ FLSs in the synovial tissue of OA patients and clinical parameters. The results showed that both the Kellgren–Lawrence grade and pain score were positively correlated with the ratio of HMGCR^+^ FLSs (Figure S1A,B). Furthermore, immunofluorescence staining revealed a higher abundance of HMGCR^+^ p16^+^ cells in the synovium of both OA patients and OA mice, compared to their respective normal groups (Figure 2F,G). Our findings indicate a positive correlation between the percentage of HMGCR^+^ FLSs and the senescence of FLSs.

### 2.3 Inhibition of HMGCR In Vitro Alleviates Cellular Senescence in Fibroblasts

To further verify the function of HMGCR in cellular senescence, we established a induction of senescence by double treatment of TNF‐α (dTNF‐InSen) cellular model in vitro by stimulating the NIH‐3T3 cell line with TNF‐α, modified from a previous study (Figure 3A) [31]. The dTNF‐InSen NIH‐3T3 cells showed upregulated expression of senescence‐associated proteins, including p16, MMP13, and IL‐6 (Figure 3B and Figure S2A), and a significant increase in the proportion of cells in the G1 phase (Figure 3C). In addition, the expression levels of HMGCR and other senescence‐associated genes were increased in dTNF‐InSen model (Figure 3D). Immunofluorescent staining showed a high expression of HMGCR in dTNF‐InSen FLSs (Figure 3E).

**FIGURE 3:**
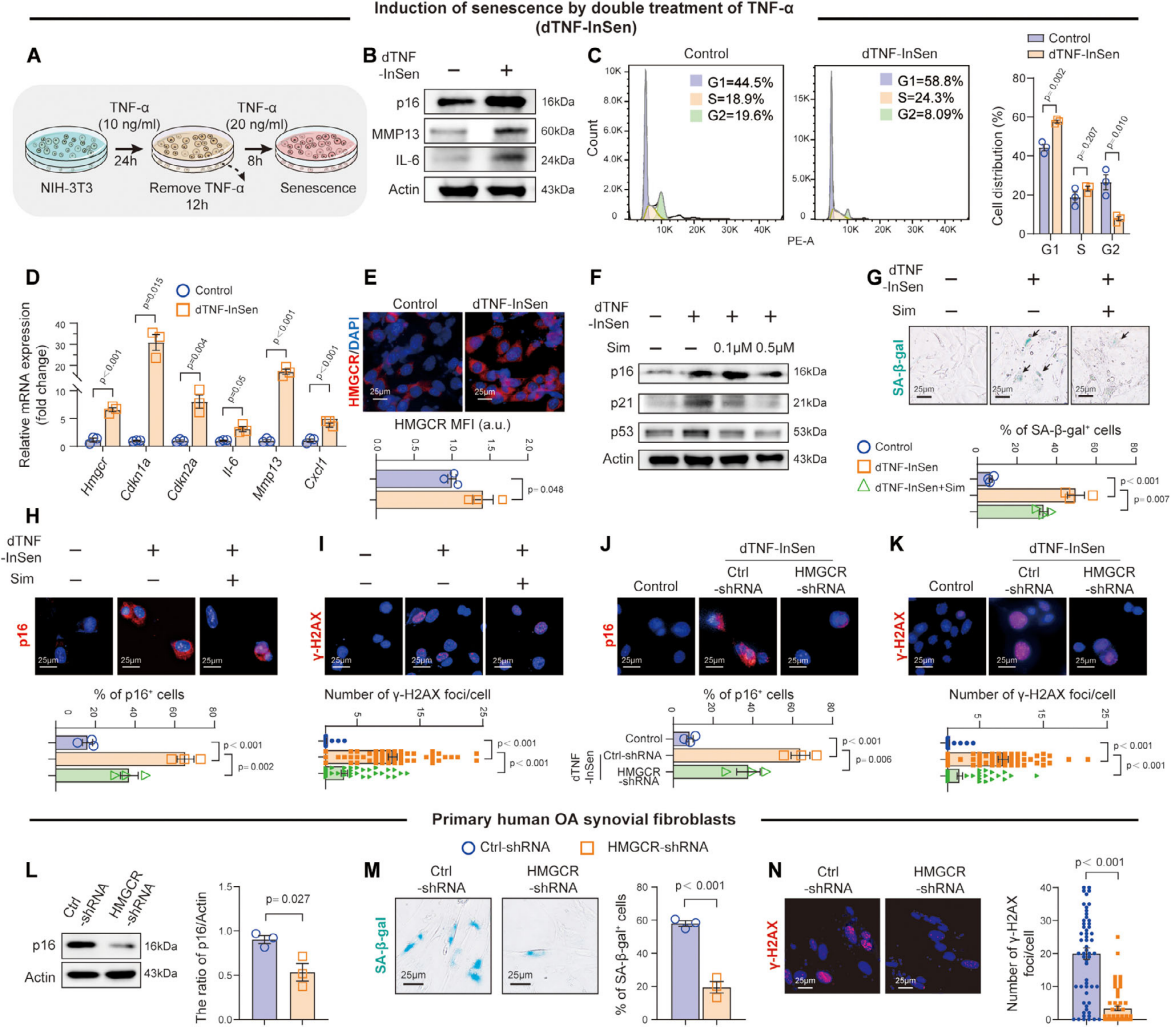
HMGCR inhibition reverses cellular senescence of FLSs in vitro. (A) Schematic diagram of dTNF‐InSen model in NIH‐3T3 cells. Briefly, NIH‐3T3 cells were treated with 10 ng mL^−1^ TNF‐α for 24 h, followed by fresh culture medium without TNF‐α for 12 h. Then, the cells were treated with 20 ng mL^−1^ TNF‐α for 8 h to establish the dTNF‐InSen model. (B) Immunoblot analysis of p16, MMP13, and IL‐6 protein levels in dTNF‐InSen FLSs. (C) Analysis of cell cycle distribution by flow cytometry in dTNF‐InSen NIH‐3T3 cells. (D) mRNA expression of HMGCR and senescence‐associated genes in dTNF‐InSen model. *n* = 3 per group. (E) Immunofluorescence staining of HMGCR expression and quantification of mean HMGCR MFI in dTNF‐InSen model. *n* = 3 per group. (F) Immunoblot analysis of cellular senescence markers in dTNF‐InSen FLSs following treatment with different concentrations of Sim. (G) Representative SA‐β‐gal staining and quantitation of SA‐β‐gal^+^ cells following treatment with 0.5 µm Sim in the dTNF‐InSen FLSs. *n* = 3 per group. (H,I) Typical immunofluorescence staining images and quantitative analysis of p16 (*n* = 3 per group) and γ‐H2AX (*n* = 60 per group) protein expression in the dTNF‐InSen FLSs after treatment with 0.5 µm Sim. (J,K) Representative immunofluorescence staining images and quantitative analysis of p16 (*n* = 3 per group) and γ‐H2AX (*n* = 60 per group) protein expression in the dTNF‐InSen FLSs following the transfection of the HMGCR‐shRNA or Control (Ctrl)‐shRNA plasmid. In the control group, cells were transfected with Ctrl‐shRNA plasmid without dTNF‐InSen treatment. Following HMGCR knockdown in primary human OA synovial fibroblasts using HMGCR‐shRNA, (L) the immunoblot analysis of p16, (M) the activity of SA‐β‐gal, and (N) the numbers of γ‐H2AX foci per cell were performed. All statistical tests were two‐sided. Student's *t*‐test (C,D (HMGCR, Cdkn2a, Mmp13, Cxcl1), E,L,M). Welch's *t*‐test (D (Cdkn1a)). Mann–Whitney *U* (D (Il‐6)). One‐way ANOVA with Bonferroni test (G,H,J). Fisher's exact test (I,K,N). dTNF‐InSen, induction of senescence by double treatment of TNF‐α; MFI, fluorescence intensity; Sim, simvastatin.

Next, simvastatin (a competitive inhibitor of HMGCR) was used to evaluate the role of HMGCR in cellular senescence. Simvastatin treatment decreased the expression of senescence‐associated proteins in dTNF‐InSen FLSs, including p16, p21, and p53 (Figure 3F and Figure S2B). β‐galactosidase is another biomarker of cellular senescence [21]. Simvastatin treatment decreased Senescence‐Associated β‐galactosidase (SA‐β‐gal) activity in dTNF‐InSen FLSs (Figure 3G). Additionally, immunofluorescent staining showed that simvastatin decreased the proportion of p16^+^ cells in dTNF‐InSen FLSs (Figure 3H). A further hallmark of cellular senescence is nuclear DNA damage, which consists of amplified chromatin‐remodeling events like histone H2AX phosphorylation (γ‐H2AX) [32]. The expression of γ‐H2AX was also inhibited by simvastatin treatment in dTNF‐InSen FLSs (Figure 3I). To further verify the effect of HMGCR on cellular senescence, we knocked down HMGCR using a plasmid‐based short hairpin RNA (shRNA) in vitro. The knockdown efficiency was validated by detecting the protein expression level of HMGCR (Figure S2C). The expression levels of p16 and γ‐H2AX were significantly reduced after transfection with shRNA‐2 in dTNF‐InSen FLSs, compared to the control group (Figure 3J,K).

Considering that NIH‐3T3 mouse embryonic fibroblast cell line differs to some extent from human primary FLSs, we performed additional experiments using primary human OA synovial fibroblasts (OA‐FLSs). Three additional shRNA plasmids targeting human HMGCR were generated, and their knockdown efficiency was evaluated in OA‐FLSs (Figure S2D). Senescent phenotypes of OA‐FLSs were reversed following HMGCR knockdown by shRNA‐3, as evidenced by reduced p16 expression, SA‑β‑gal activity, and γ‑H2AX foci formation (Figure 3L–N). Besides, we evaluated the senescent phenotype of HMGCR^+^ FLSs by immunofluorescence double‐staining analysis of HMGCR and p16 in OA‐FLSs. Compared with HMGCR^−^ cells, HMGCR^+^ cells showed a higher proportion of p16^+^ cells (Figure S2E). Collectively, these results indicate that suppression of HMGCR attenuates senescence‐associated phenotypes of FLSs in vitro.

### 2.4 HMGCR‐Mediated Cholesterol Biosynthetic Metabolism Accelerates Fibroblast Senescence

To analyze metabolic signatures in HMGCR‐regulated senescence, liquid chromatography–mass spectrometry (LC–MS)‐based untargeted metabolomics was performed on HMGCR‐knockdown senescent cells (Figure 4A). We divided NIH‐3T3 cells into two groups: Group A was transfected with the control plasmid, while Group B was transfected with the shHMGCR plasmid. Subsequently, both groups were treated with TNF‐α to induce cellular senescence. Alterations in 800 metabolites were identified, and differential abundance analysis of metabolic pathways was performed to compare the two groups. Kyoto Encyclopedia of Genes and Genomes (KEGG) analysis revealed prominent alterations in several pathway, including amino acid, cofactor/vitamin, and particularly lipid metabolism pathways (Figure 4B). Hierarchical clustering was performed on the top 10 metabolites that showed the most significant differences between the groups (Figure 4C). Interestingly, cholesterol sulfate (CS), a downstream metabolite of cholesterol, was significantly downregulated in HMGCR‐knockdown senescent cells (Figure 4D). A study reported that CS could activate the sterol regulatory element‐binding protein 2 (SREBP2) and promote cholesterol biosynthesis [33]. Nile Red staining indicated that cholesterol was elevated in the synovium of OA patients and DMM‐induced OA mice, compared with the control groups (Figure S3A,B). Notably, cholesterol in dTNF‐InSen FLSs was reduced after treatment with either simvastatin or HMGCR‐shRNA vector (Figure S3C,D). Since the accumulation of cholesterol could trigger cellular senescence [27], we speculated that CS may contribute to senescent phenotypes in the dTNF‐InSen model. Next, we treated FLSs with exogenous CS. The results showed that CS triggered cellular senescence, including elevated the percentages of p16^+^ cells and the number of γ‐H2AX foci per cell, which were rescued by simvastatin (Figure 4E,F). Additionally, methyl‐β‐cyclodextrin (MβCD) can be used to deplete cholesterol [26]. The cellular senescent phenotypes of dTNF‐InSen cells were also reversed by MβCD treatment (Figure 4G,H). Together, the above data suggest that HMGCR‐mediated cholesterol biosynthetic metabolism contributes to cellular senescence in FLSs.

**FIGURE 4:**
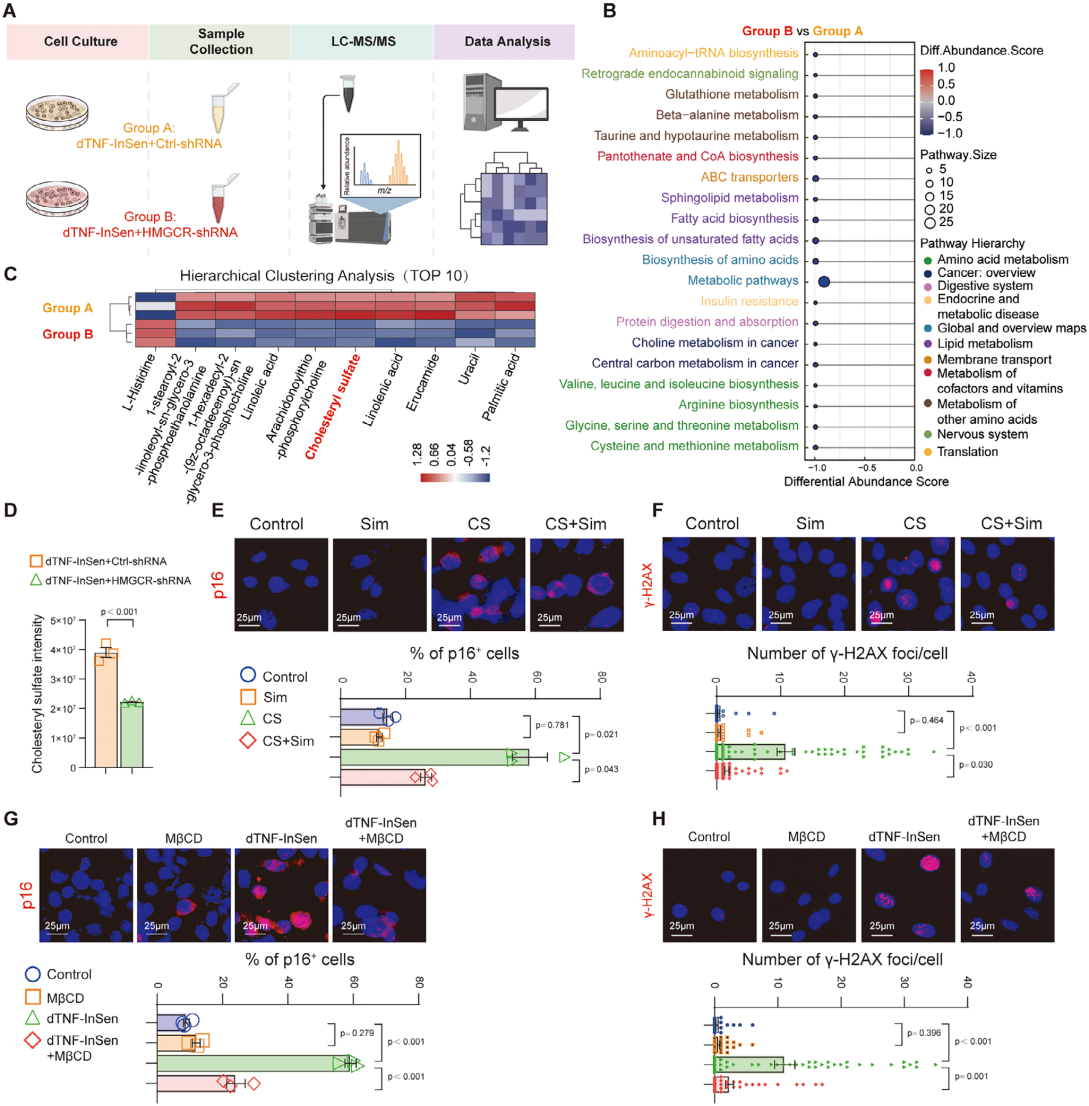
The HMGCR‐dependent cholesterol biosynthesis pathway contributes to fibroblast senescence. (A) Workflow of metabolomic analyses. NIH‐3T3 cells were divided into two groups, dTNF‐InSen+Ctrl‐shRNA and dTNF‐InSen+HMGCR‐shRNA, which were transfected with plasmids expressing control shRNA or HMGCR shRNA, respectively. Then, the cells were treated with TNF‐α to induce senescence. Cell lysates were collected and analyzed by LC–MS/MS untargeted metabolomics. (B) KEGG pathway differential abundance score of the untargeted metabolomics between the two groups. The lipid metabolism pathways are shown in purple. (C) Hierarchical clustering analysis of metabolites displaying the top 10 metabolites ranked by significance of difference. (D) Quantification of CS levels in two groups from LC–MS/MS untargeted metabolomics data. *n* = 3 per group. (E,F) Representative immunofluorescence images and the corresponding quantitative analysis of p16 (*n* = 3 per group) and γ‐H2AX (*n* = 60 per group). NIH‐3T3 cells were treated with 25 µm CS for 2 days, alone or in combination with 0.5 µm Sim. (G,H) Representative immunofluorescence images and the corresponding quantitative analysis of p16 (*n* = 3 per group) and γ‐H2AX (*n* = 50 per group). NIH‐3T3 cells were pretreated with 0.25% (w/v) MβCD for 1 h before TNF‐α treatment, with continued MβCD exposure during the final 8 h of the dTNF‐InSen model. All statistical tests were two‐sided. Student's *t*‐test (D). One‐way ANOVA with Tamhane's T2 test (E,G). Fisher's exact test (F,H). CS, cholesterol sulfate; dTNF‐InSen, induction of senescence by double treatment of TNF‐α; Sim, simvastatin; MβCD, methyl‐β‐cyclodextrin.

### 2.5 Activation of AKT Enhances the Expression of HMGCR in Fibroblasts

KEGG analysis of scRNA‐seq data (Figure 2A) revealed a significant enrichment of the PI3K/AKT and ERK1/2 signaling pathways in HMGCR^+^ FLSs of OA mice. These pathways have been previously implicated in cellular senescence [34, 35]. Specific inhibitors targeting the PI3K/AKT (LY294002), ERK1/2 (PD0325901), JNK (SP600125), p38 MAPK (SB203580), and ERK5/BMK1 (XMD8‐92) pathways were used to verify the role of the corresponding signaling pathways in upregulated HMGCR expression during cellular senescence (Figure 5A). Inhibition of the PI3K/AKT pathway led to a marked reduction in HMGCR transcript levels in dTNF‐InSen FLSs (Figure 5B). We then generated a recombinant luciferase reporter plasmid, HMGCR (WT)‐Luc, harboring the HMGCR promoter region (−2000 to +100 bp). Luciferase reporter assay subsequently confirmed that HMGCR transcription was significantly increased in dTNF‐InSen FLSs and that this increase was abolished by the PI3K/AKT inhibitor (Figure 5C). Meanwhile, the PI3K/AKT inhibition suppressed HMGCR protein levels in dTNF‐InSen FLSs, as well as senescence‐associated proteins such as p16, p21, and p53 (Figure 5D and Figure S4). Immunofluorescence staining for p16 and γ‐H2AX further corroborated these findings (Figure 5E,F). Collectively, these data indicate that PI3K/AKT pathway activation upregulates HMGCR and contributes to TNF‐α‐induced cellular senescence.

**FIGURE 5:**
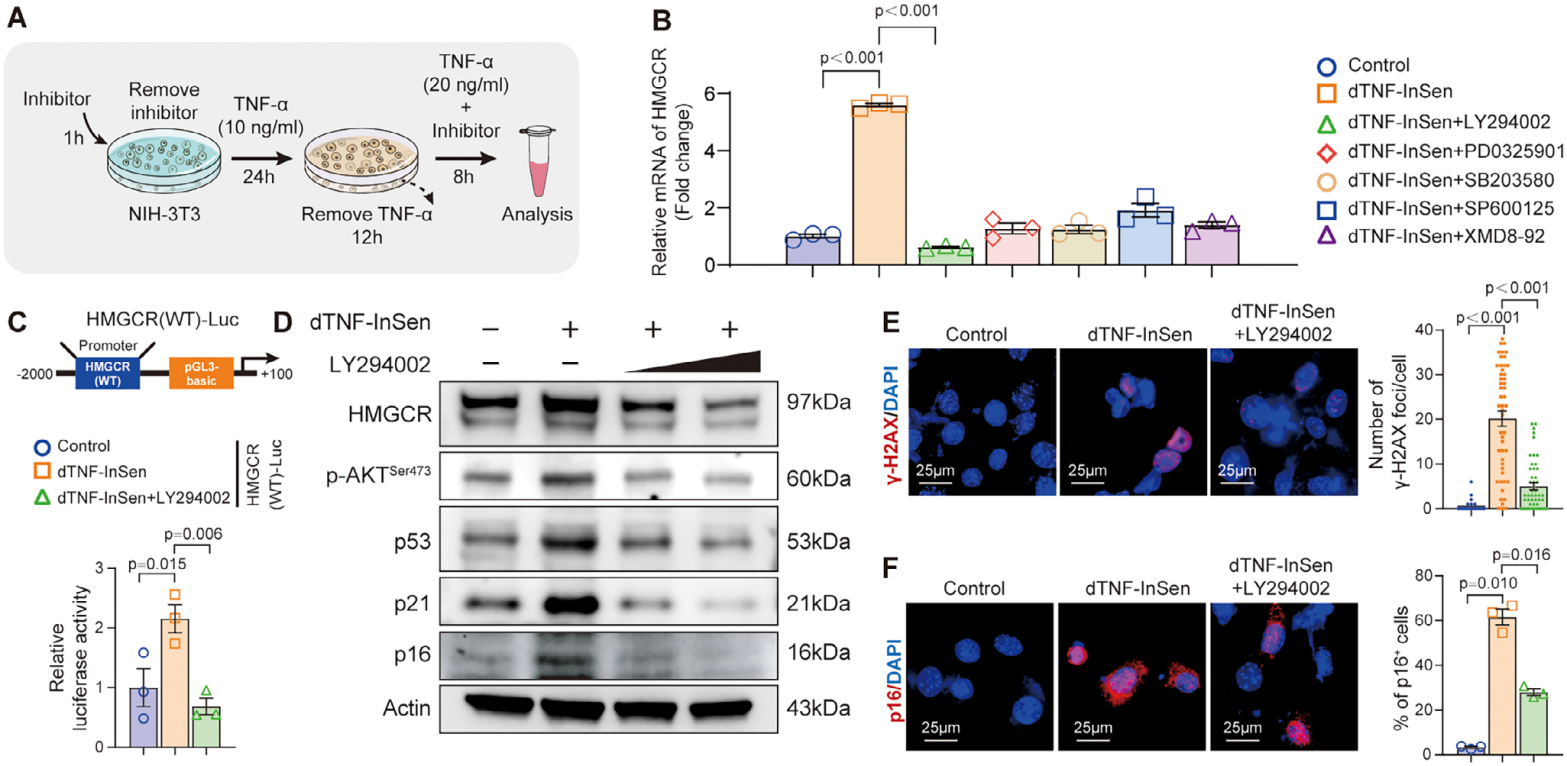
AKT phosphorylation is linked to HMGCR‐mediated senescence in fibroblasts. (A) Schematic of the inhibitor‐treatment strategy in dTNF‐InSen model. Briefly, NIH‐3T3 cells were pretreated with inhibitors for 1 h, and the inhibitors were maintained for the last 8 h during TNF‐α‐induced senescence. Inhibitors were applied at the following concentrations: 100 µm LY294002 (PI3K‐AKT pathway), 0.5 µm PD0325901 (ERK1/2 pathway), 5 µm SB203580 (p38 MAPK pathway), 20 µm SP600125 (JNK pathway), and 2 nm XMD8‐92 (ERK5/BMK1 pathway). (B) HMGCR mRNA was analyzed by quantitative real‑time polymerase chain reaction (RT‐qPCR) following treatment with inhibitors of different pathways in dTNF‐InSen model. *n* = 3 per group. (C) Schematic of dual‐luciferase reporter (HMGCR (WT)‐Luc) construct containing the HMGCR promoter region (−2000 to +100). Quantification of relative luciferase activity of HMGCR in senescent NIH‐3T3 FLSs with or without treatment of PI3K‐AKT inhibitor LY294002. *n* = 3 per group. (D) Immunoblot analysis of specified proteins following treatment with graded concentrations of LY294002 in the dTNF‐InSen FLSs. (E) Representative immunofluorescence staining images and quantification of numbers of γ‐H2AX foci per cell. *n* = 50 per group. (F) Representative images and quantification of the percentage of p16^+^ cells following different treatments. *n* = 3 per group. All statistical tests were two‐sided. One‐way ANOVA with Bonferroni test (B,C). One‐way ANOVA with Tamhane's T2 test (F). Fisher's exact test (E). dTNF‐InSen, induction of senescence by double treatment of TNF‐α.

### 2.6 Activation of AKT1 Increases Its Binding to Insig1 at Lys140

The transcription of HMGCR is regulated via the classic sterol‐mediated negative feedback, primarily through the SCAP–SREBP2 axis. In brief, SCAP is defined as the protein that senses intracellular sterol levels and exists in a complex with SREBP2. In response to high levels of oxysterols in cells, the SCAP–SREBP2 complex binds to Insig1/2 proteins and the Insig–SCAP–SREBP2 complex is retained in the endoplasmic reticulum (ER), blocking cleavage of SREBP2 and subsequent HMGCR transcription [36]. Moreover, AKT activation could phosphorylate Insig1/2, thereby suppressing transcription of the downstream Hmgcr gene in human hepatocellular carcinoma cells [37]. Thus, we speculate that the activation of AKT1 upregulates HMGCR in senescent FLSs through its interaction with Insig1.

Co‐immunoprecipitation (Co‐IP) analyses revealed that AKT1 co‐precipitated with Insig1 in the NIH‐3T3 model (Figure 6A). Subsequently, protein–protein docking analysis revealed a potential direct physical interaction between AKT1 and Insig1 in the native state (Figure 6B and Figure S5A) and the analysis of the contact surface and hydrogen bond interactions in the AKT1–Insig1 complex is detailed in Tables S1 and S2. As phosphorylation at the Ser473 and Thr308 residues is essential for the activation of AKT1 [38], computational modeling of phosphorylation at these two sites was conducted to simulate the activation of AKT1. To show conformational changes in the AKT1–Insig1 complex following AKT activation, we conducted 100 ns molecular dynamics simulations comparing the nonphosphorylated (AKT1–Insig1) and fully phosphorylated (pAKT1–Insig1) states (Figure S5B,D). Both the AKT1–Insig1 and pAKT1–Insig1 complexes achieved equilibrium and convergence during the 20–80 ns and 15–100 ns periods, respectively, which confirmed that the two complexes maintained their structural stability during the molecular dynamics (MD) simulation (Figure 6C and Figure S5E,F). To further evaluate the relative binding free energy between AKT1 and Insig1 for the two different states, Δ*G*_bind_ was calculated using the molecular mechanics/Poisson–Boltzmann surface area (MM/PBSA) method, which are listed in Tables S3 and S4. We found that the binding affinity between AKT1 and Insig1 in the phosphorylated state was significantly higher than that in the nonphosphorylated state (Figure 6D). Furthermore, 2D free energy landscapes (FEL) were generated for each complex, with the radius of gyration (*R*_g_) and RMSD serving as the collective variables (Figure 6E and Figure S5C,G). The FEL revealed the energetic states of the complexes of AKT1–Insig1 and pAKT1–Insig1 over the 0–100 ns simulation. The darker regions on the FEL indicated lower free energy and higher complex stability. Representative structures from the deep basins were selected as models for subsequent investigations. Detailed information on hydrogen bonds at various positions within the two complexes is provided in Tables S5 and S6. Protein–protein interactions in those complexes were visualized as hydrogen bonds and hydrophobic interactions (Figure 6E and Figure S5H,I). The structural analysis confirmed that AKT1 at Ser473 was located at the binding interface with Insig1. Importantly, a hydrogen bond with Insig1 at Lys140 is consistently maintained in both the nonphosphorylated and phosphorylated states. We hypothesize that Lys140 of Insig1 and Ser473 of AKT1 are essential for the stability of the AKT1–Insig1 complex following AKT activation.

**FIGURE 6:**
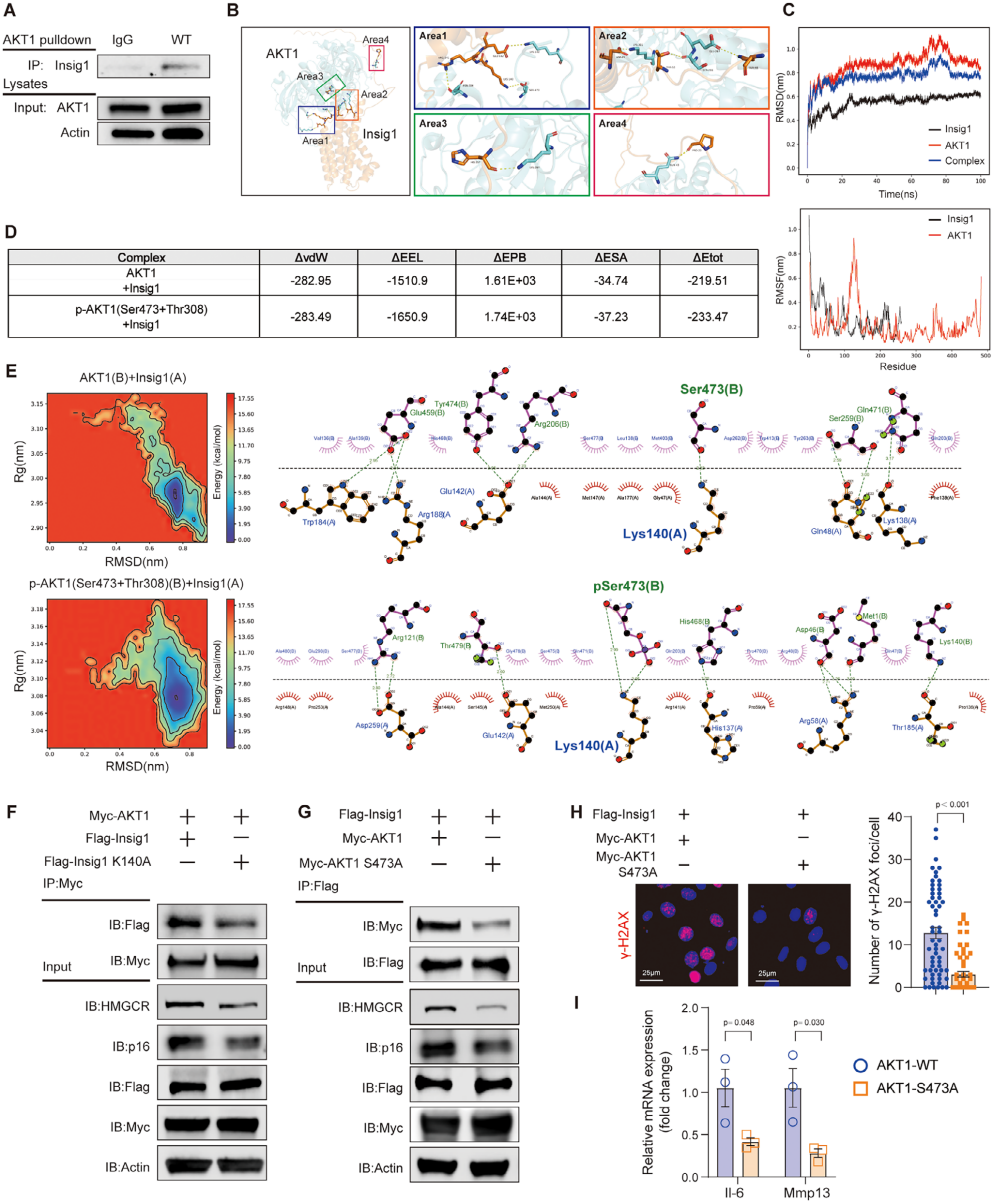
Phosphorylation of AKT1 at Ser473 enhances its binding to Lys140 of Insig1. (A) The interaction between endogenous AKT1 and Insig1 in NIH‐3T3 cells was demonstrated by co‐immunoprecipitation. (B) The molecular docking mode of the interaction between AKT1 and Insig1. (C) RMSD (up) and RMSF (down) analysis showing the binding of AKT1 and Insig1. Red, black, and blue represent AKT1, Insig1, and the complex, respectively. (D) Molecular mechanics‐Poisson‐Boltzmann surface area (MM‐PBSA) analysis of AKT1–Insig1 and pAKT1 (Ser473+Thr308)–Insig1 complexes. Calculation of energy terms (kcal per mol) from the 100 ns trajectory. Van der Waals Energy (ΔvdW) indicated nonpolar interactions between molecules. Electrostatic Energy (ΔEEL) indicated electrostatic interaction energy between charges. Polar solvation energy (ΔEPB) represents the solvation energy of molecules in polar solvents, calculated by employing the Poisson–Boltzmann equation. Nonpolar Solvation Energy (ΔESA) was typically calculated based on the solvent‐accessible surface area multiplied by an empirical coefficient. Δ*G*_bind_ (Δ*E*_tot_) represented the overall binding affinity between interacting molecules. (E) FEL of the two complexes, constructed using RMSD and *R*_g_ as variables, displaying the energy distribution in different conformational states between 0 and 100 ns (left). H‐bonds and hydrophobic interactions by LigPlus illustrating interactions between AKT1 and Insig1 in two different states, which are displayed in green dotted lines and red or pink spikes, respectively. The main panel shows hydrogen bonds and hydrophobic interactions surrounding AKT1 Ser473 and pSer473 with Insig1 (right). (F,G) Co‐IP analysis to assess AKT1–Insig1 interaction in the dTNF‐InSen NIH‐3T3 cells after transfection of Insig1 K140A vector under conditions of AKT1 WT overexpression or AKT1 S473A vector under the condition of Insig1 WT overexpression. (H,I) The expression of γ‐H2AX (*n* = 60 per group), Il‐6 (*n* = 3 per group), and Mmp13 (*n* = 3 per group) in the dTNF‐InSen FLSs after the transfection of AKT1 WT or AKT1 S473A vector under the condition of Insig1 WT overexpression, compared to the controls. All statistical tests were two‐sided. Fisher's exact test (H). Student's *t*‐test (I). dTNF‐InSen, induction of senescence by double treatment of TNF‐α; RMSD, protein backbone root mean square deviation; RMSF, root mean square fluctuation; FEL, free energy landscape; *R*_g_, radius of gyration.

Subsequently, mutations of Lys140 in Insig1 to alanine (Insig1 K140A) and Ser473 in AKT1 to alanine (AKT1 S473A) were constructed (Figure S6A–D). Co‐IP analysis suggested AKT1–Insig1 interaction was decreased in dTNF‐InSen FLSs after transfection with Insig1 K140A under conditions of AKT1 WT overexpression, compared to the wild‐type control. Moreover, the expression of HMGCR also showed the corresponding decrease in the Insig1 K140A group (Figure 6F and Figure S6E). To further investigate the impact of AKT1 activation on its interaction with Insig1, we transfected the AKT1 S473A or AKT1 WT plasmids into dTNF‐InSen FLSs under the condition of Insig1 WT overexpression. Co‐IP results showed the binding affinity between AKT1 and Insig1 was decreased in dTNF‐InSen model following mutation of AKT1 at Ser473 (Figure 6G). Additionally, HMGCR and p16 expression was decreased in AKT1 S473A group (Figure 6G). The dTNF‐InSen FLSs in AKT1 S473A group exhibited the decreased expression of senescence‐associated markers, including γ‐H2AX, Il‐6, and Mmp13 (Figure 6H,I). Collectively, phosphorylation of AKT1 at Ser473 enhances its binding to Lys140 of Insig1, which influences formation of the AKT1–Insig1 complex, the expression of HMGCR, and cellular senescence.

### 2.7 Insig1–SCAP–SREBP2 Mediates AKT Activation‐Induced HMGCR Upregulation in dTNF‐InSen FLSs

Next, we investigated the changes in the Insig1–SCAP–SREBP2 pathway in dTNF‐InSen FLSs. The results showed that the interaction between Insig1 and SCAP was reduced in the dTNF‐InSen model (Figure 7A). Since the mature nuclear form of SREBP2 (nSREBP2) requires translocation of the SCAP–SREBP2 complex from the ER to the Golgi, we used the translocation inhibitor (PF‐429242) in the dTNF‐InSen model. The findings indicated that treatment with PF‐429242 decreased HMGCR transcription in dTNF‐InSen FLSs (Figure 7B). As mature SREBP2 binds to the SRE within the HMGCR promoter to promote HMGCR transcription [39], we engineered two recombinant luciferase reporter plasmids with SRE sequence mutations, named HMGCR‐Luc (SRE1‐Mut) and HMGCR‐Luc (SRE2‐Mut). After transfections with these plasmids in dTNF‐InSen FLSs, HMGCR luciferase activity decreased compared with the control group (Figure 7C). Moreover, we designed two primer sets targeting HMGCR (containing SRE site) and HMGCR (negative control) for the following chromatin immunoprecipitation‐quantitative polymerase chain reaction (PCR) (ChIP‐qPCR) assay. Compared with the negative control group, the binding of SREBP2 to the SRE‐containing region of the HMGCR promoter was enriched in dTNF‐InSen FLSs (Figure 7D). These data suggest that the upregulation of HMGCR in dTNF‐InSen FLSs is partially mediated by the Insig1–SCAP–SREBP2 axis.

**FIGURE 7:**
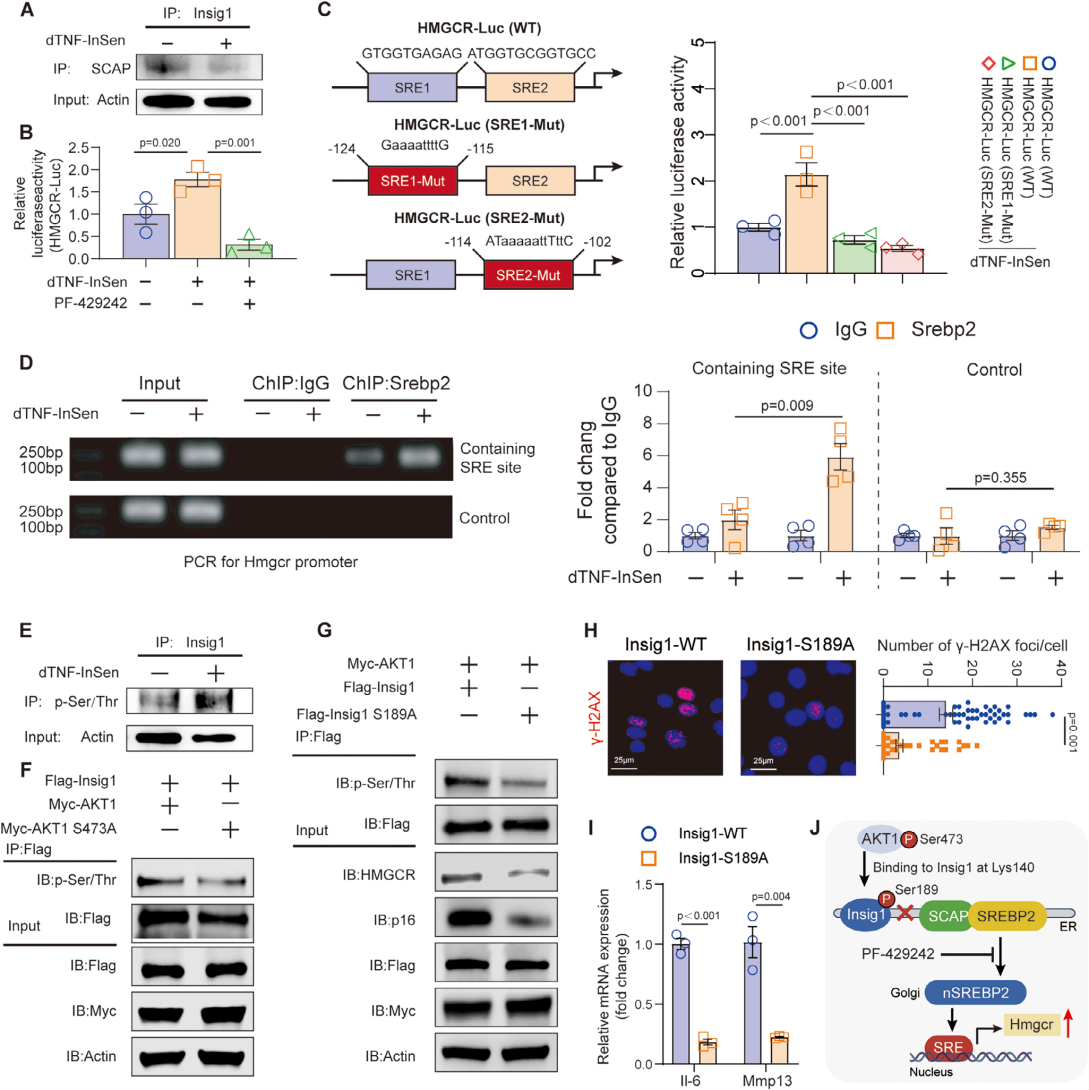
AKT1 phosphorylation enhances the transcriptional expression of HMGCR partially through the dissociation of Insig1 from SCAP. (A) Co‐IP assay to validate the interaction between endogenous Insig1 and SCAP during TNF‐α‐induced senescence in NIH‐3T3 cells. (B) Luciferase reporter assay to quantify the HMGCR WT luciferase activity after pretreating with 10 nm PF‐429242 for 1 h, with PF‐429242 maintained for the last 8 h in the dTNF‐InSen NIH‐3T3 cells. *n* = 3 per group. (C) Diagrams of HMGCR reporter vectors containing the wild‐type SRE1 and SRE2 sequences (HMGCR‐Luc (WT)), the mutated SRE1 sequences (HMGCR‐Luc (SRE1‐Mut)), and the mutated SRE2 sequences (HMGCR‐Luc (SRE2‐Mut)). Uppercase and lowercase letters represent the wild‐type and mutant sequences, respectively (left). Luciferase reporter assay to quantify the HMGCR luciferase activity in dTNF‐InSen FLSs transfected with three plasmids (right). *n* = 3 per group. (D) ChIP‐qPCR. NIH‐3T3 cells were induced to senescence by double treatment of TNF‐α. ChIP was performed against SREBP2. The SREBP2 ChIP complex was used for PCR to amplify the predicted promoter regions. (E) Co‐IP assay to assess serine/threonine phosphorylation of Insig1 in the dTNF‐InSen FLSs. (F,G) Co‐IP assay to assess serine/threonine phosphorylation of Insig1 in the dTNF‐InSen FLSs after transfection of Insig1 S189A or AKT1 S473A vector, compared to the wild‐type controls. (H,I) The expression of γ‐H2AX (*n* = 60 per group), Il‐6 (*n* = 3 per group), and Mmp13 (*n* = 3 per group) in the dTNF‐InSen FLSs after transfection of Insig1 WT or Insig1 S189A vector under the condition of AKT1 WT overexpression, compared to the controls. (J) The working model for upregulated HMGCR expression in senescent FLSs by AKT1–Insig1–SCAP–SREBP2 pathway. All statistical tests were two‐sided. One‐way ANOVA with Bonferroni test (B,C). Student's *t*‐test (D,I). Fisher's exact test (H). dTNF‐InSen, induction of senescence by double treatment of TNF‐α. PF‐429242, an inhibitor of SCAP–SREBP2 translocation.

A previous study has shown that phosphorylation of Insig1 could promote its dissociation from the SCAP–SREBP2 complex [37]. We hypothesize that AKT1 activation may influence Insig1 phosphorylation in senescent FLSs. Co‐IP results showed an increased phosphorylation of Insig1 in dTNF‐InSen FLSs (Figure 7E). To suppress the activation of AKT1 in the senescence model, we transfected the AKT1 S473A plasmid into dTNF‐InSen FLSs. The results showed that the increased phosphorylation of Insig1 in senescent FLSs may be closely associated with AKT1 activation (Figure 7F). Based on previous study, AKT could phosphorylate human Insig1 at Ser207, and the Ser207 residue in human Insig1 was homologous to Ser189 in mouse Insig1 [37]. Thus, a mutation of Ser189 in Insig1 to alanine (Insig1 S189A) was constructed (Figure S6F). Compared to the wild‐type control, the phosphorylation of Insig1 was reduced in dTNF‐InSen cells after transfection of Insig1 S189A vector under the condition of AKT1 WT overexpression, as well as the decreased expression of HMGCR and p16 (Figure 7G). Additionally, dTNF‐InSen FLSs in Insig1 S189A group displayed the decreased expression of γ‐H2AX, IL‐6, and MMP13 (Figure 7H,I). These data suggest that Ser189 of Insig1 is phosphorylated by AKT1 activation in dTNF‐InSen FLSs and Insig1 phosphorylation may modulate downstream HMGCR expression and senescence‐marker expression. Taken together, activated AKT1 may promote phosphorylation of Insig1 at Ser189, potentially enhancing the dissociation of Insig1 from SCAP–SREBP2 complex, thereby contributing to SREBP‐mediated HMGCR transcription via the SRE‐containing promoter region in dTNF‐InSen FLSs (Figure 7J).

### 2.8 Targeted Inhibition of HMGCR in Synovial FLSs Alleviates OA Progression in DMM‐Induced OA Mice

To evaluate the role of HMGCR in OA progression and FLSs senescence, we constructed AAV9‐shHMGCR modified to display HAP‐1, a reported synovial‐FLS‐targeting peptide (Figure 8A) [23, 24]. Additionally, using scRNA‐seq data from OA mouse synovium (GSE211584), we identified pronounced Fap expression specifically in FLSs and most notably in senescent FLSs (Figure 8B). Ultimately, the AAV vector was engineered to include an Fap promoter, which was designed to facilitate improved targeting of synovial FLSs by intra‐articular injection (Figure 8C). We measured serum cholesterol and LDL‐C levels in experimental animals. The results showed no significant change in systemic lipid levels after intra‐articular injection of AAV‐shHMGCR, compared to the control group (Figure S7A,B). To assess the off‐target effects of the AAVs, we measured EGFP fluorescence intensity using an in vivo imaging system in individual tissues and organs (heart, liver, spleen, lung, kidney, and knee joint) harvested from mice that received intra‐articular injections of AAV‐Ctrl or AAV‐shHMGCR. The fluorescence signal was predominantly localized to the knee joint and partially in the liver (Figure S7C,D,), which was consistent with a previous study [23]. Immunofluorescence staining further indicated that abundant of EGFP‐positive cells were present in the synovium of mice after intra‐articular injection of AAV (Figure 8D). Additionally, hematoxylin and eosin (H&E) staining revealed that neither AAV‐Ctrl nor AAV‐shHMGCR resulted in pathological injury in the examined tissues and organs (Figure S7E).

**FIGURE 8:**
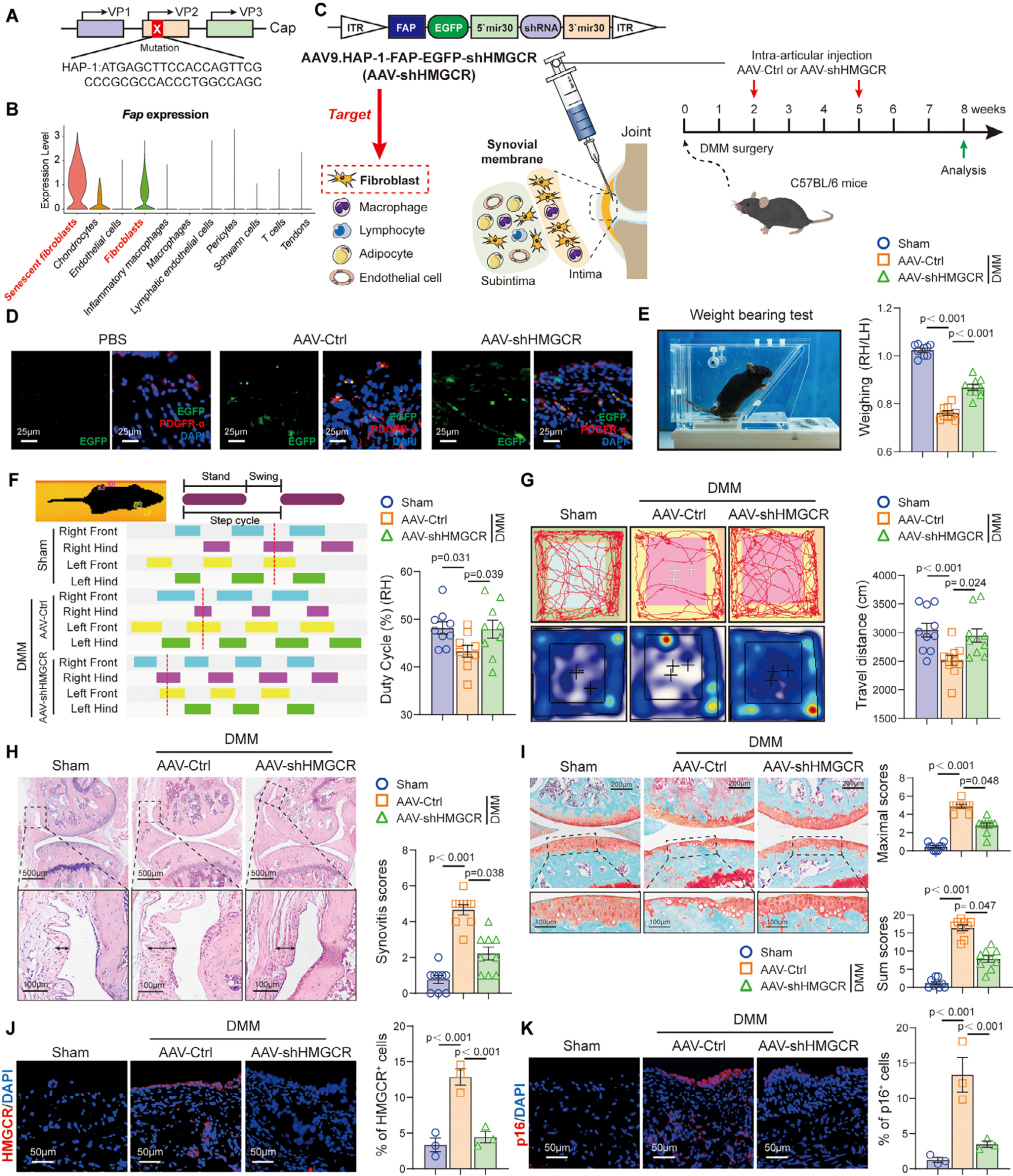
Knockdown of HMGCR in synovial FLSs alleviates the behavioral and pathological progression in DMM‐induced OA mice. (A) Schematic of designed synovium‐specific AAV capsid. The synovium‐targeting peptide HAP‐1 was inserted into the N‐terminus of VP2 in the AAV9 capsid. (B) Violin plot showing Fap expression in different synovial cell types from OA mice (data from GSE211584). (C) Schematic diagrams of HMGCR‐specific knockdown strategy in FLSs using a synovium‐targeted AAV9.HAP‐1 vector carrying Fap‐driven HMGCR shRNA labeled with EGFP (left). Experimental design for the animal studies (right). The intra‐articular injection of 10 µL AAV9 (1 × 10^1^ ^1^ GC) carrying either HMGCR shRNA or a negative control was administered at the second and fifth weeks after DMM surgery. Behavioral tests and pathological examination were conducted at 8 weeks after DMM. (D) Fluorescence microscope images showing costaining of EGFP and PDGFR‐α in mouse knee joints following intra‐articular injection of AAV‐Ctrl or AAV‐shHMGCR. (E) Static weight bearing test assessing hindlimb weight distribution (RH/LH) in mice after different treatments. *n* = 9 per group. (F) CatWalk gait analysis evaluating the Duty Cycle (%) index of Right Hindlimb in different groups. *n* = 9 per group. (G) Open field test evaluating the travel distance during a 5 min session in the chamber. *n* = 9 per group. (H) Representative images of H&E staining with synovial inflammation indicated by dashed double‐headed arrows (left) and the extent of synovial inflammation quantified by the synovitis score (right) across the three groups. *n* = 9 per group. (I) Safranin O/fast green staining of cartilage morphology (left) and the Osteoarthritis Research Society International (OARSI) scoring system quantifying the severity of cartilage damage in the three groups (right). *n* = 9 for per group. (J,K) Representative immunofluorescence staining images and quantification of p16^+^ and HMGCR^+^ cells in mouse synovium across groups. *n* = 3 per group. Data are presented as the mean ± standard error of the mean (SEM). All statistical tests were two‐sided. One‐way ANOVA with Bonferroni test (E,F,G,J,K). Kruskal–Wallis test (H,I (maximal scores and sum scores)). RH, right hindlimb; LH, left hindlimb.

We then evaluated whether intra‐articular injection of AAV‐shHMGCR could exert therapeutic effects in DMM‐induced OA mice. Assessment of postural equilibrium revealed that weight‐bearing capacity was markedly improved in the AAV‐shHMGCR group, compared with the AAV‐Ctrl group (Figure 8E). CatWalk gait analysis revealed that intra‐articular injection of AAV‐shHMGCR significantly increased the duty cycle of the injured right hindlimb in OA mice, compared to the AAV‐Ctrl group (Figure 8F). Next, the open field test, another behavioral experiment, was used to evaluate locomotor activity. The results demonstrated that intra‐articular injection of AAV‐shHMGCR in OA mice resulted in an increased travel distance, compared to the AAV‐Ctrl group (Figure 8G). Furthermore, we found that treatment with AAV‐shHMGCR resulted in a significant mitigation of DMM‐induced synovitis and cartilage destruction in OA mice (Figure 8H,I). Immunofluorescence staining demonstrated a reduction in the populations of HMGCR^+^ and p16^+^ cells upon AAV‐shHMGCR treatment (Figure 8J,K). To eliminate the effect of the AAV viral vector itself, we also performed intra‐articular injection of the AAV empty‐vector (AAV‐Vector) in DMM‐induced OA mice. No significant differences were observed in weight‐bearing or open‐field tests between the AAV‐Vector group and AAV‐Ctrl group (Figure S7F,G). Pathological staining of knee joints from mice in AAV empty vector group revealed a similar severity of synovitis and cartilage degeneration, compared to the mice in the control group (Figure S7H). In summary, these data suggest that synovium‐targeted inhibition of HMGCR by AAV‐mediated shHMGCR delivery could alleviate OA development and FLSs senescence in DMM‐induced OA mice.

## 3 Discussion

Several studies have reported that activation of the AKT signaling pathway promotes cellular senescence in several pathological conditions. A reduction‐of‐function mutation in PI3K/AKT pathway led to extension of the lifespan in the model of *Caenorhabditis elegans* [40, 41]. Additionally, overexpression of AKT1 in U2OS cells exhibited a more severe senescent phenotype than control cells [42]. The knockdown of AKT1 markedly attenuated cellular senescence in a replicative‐senescence model of primary mouse embryonic fibroblasts [43]. Moreover, the PI3K/AKT/mTOR signaling pathway was also involved in OA progression, including cartilage degradation, subchondral bone dysfunction, and synovial inflammation [44]. Our results indicate that activation of AKT1 contributes to cellular senescence of FLSs, which leads to subsequent OA progression. Besides, research has shown that the activation of AKT1 in senescent cells was regulated by several upstream signaling pathways. For instance, TRIM22 could promote the degradation of PHLPP2, which activated AKT and further triggered cellular senescence in hepatocellular carcinoma [45]. In senescent endothelial cells, the assembly and activation of mTORC2 led to phosphorylation of AKT at Ser473 [46]. However, the upstream signals and detailed mechanisms of AKT1 activation in senescent synovial FLSs of OA are not clear, which needs to be explored in future studies.

AKT signaling pathway is closely related to sterol‐mediated negative‐feedback regulation of HMGCR. A previous study reported that the activation of PI3K/AKT induced by TGF‐β could cause overexpression of SREBP2 and its target gene Hmgcr in osteoarthritic chondrocytes [18]. Thyroid‐stimulating hormone promoted mature SREBP2 expression through activation of PI3K/AKT during bile acid synthesis [47]. Inhibition of AKT decreased SREBP2 activation and subsequent downregulation of HMGCR [48, 49]. However, the detailed mechanisms involving the transcriptional upregulation of HMGCR by AKT in senescent cells remains unknown. In the present study, we found that the interaction of AKT1 and Insig1 played an important role in HMGCR expression. The phosphorylation of AKT1 at Ser473 enhanced its binding to Insig1, which further increased the level of Insig1 phosphorylation in dTNF‐InSen FLSs. This phosphorylation change led to HMGCR upregulation via the SCAP–SREBP2 pathway. However, whether this effect of Insig1 phosphorylation by AKT1 is direct or indirect remains to be elucidated. A previous study revealed that AKT1 could phosphorylate Insig1 through PCK1 protein to regulate the expression of downstream genes that are involved in lipogenesis [37]. The role and mechanism of PCK1 in senescent OA FLSs require further investigation. In addition to Insig1–SCAP–SREBP2 pathway, whether other AKT1‐dependent mechanisms are involved in HMGCR expression in senescent FLSs requires further exploration.

OA is a systemic disease affecting multiple tissues, including cartilage, subchondral bone, muscle, and synovium. The roles and mechanisms of HMGCR‐mediated cholesterol homeostasis may vary across different cell types during OA progression. In chondrocytes, pharmacologic inhibition of cholesterol with lovastatin attenuated chondrocyte hypertrophy in OA‐induced Insig1/2 double‐knockout mouse model [50]. In human primary macrophages, cholesterol crystals could induce metabolic reprogramming and promote M1 polarization [51]. The accumulation of low‐density lipoprotein in synovial macrophages could promote osteophyte formation by activating the TGF‐β signaling pathway [52]. Our group recently reported that the downregulation of HMGCR in the quadriceps of OA mice impaired CoQ10 synthesis and promoted ferroptosis, contributing to the OA‐related muscle atrophy [19]. Furthermore, subchondral osteocytes could increase the uptake of cholesterol and transfer mitochondria to cartilage, which promoted cartilage inflammation and degradation [53]. The present data suggest that HMGCR‐mediated cholesterol metabolism accelerate senescence of synovial FLSs, thereby accelerating OA progression. However, CS was used only for exogenous functional validation in the current study. More studies should be performed to examine the expression of key enzymes involved in endogenous CS synthesis during the senescence process. These findings indicate that cholesterol metabolism could accelerate OA development by affecting multiple joint tissues. Besides, serum total cholesterol also affects OA phenotypes [54, 55, 56]. The results in our study show that intra‐articular injection of AAV‐shHMGCR under the given dose does not achieve a systemic lipid‐lowering effect, suggesting that abnormal cholesterol metabolism in the local joint mainly contributes to OA progression in our model.

Under normal physiological conditions, negative‐feedback regulation of HMGCR by cholesterol serves as a key mechanism for maintaining cholesterol homeostasis. However, this feedback regulation might be disrupted under certain pathological conditions. A previous study reported that constitutively methylated RIG‐I associated with AMPKα to inhibit HMGCR phosphorylation, thus maintaining a high level of HMGCR and cholesterol in nonalcoholic fatty liver disease [57]. Additionally, chaperone‐mediated autophagy deficiency in hepatocytes blocked the entry of the HMGCR protein into the lysosome, which triggered a concomitant rise in both HMGCR protein levels and cellular cholesterol content [58]. Lipopolysaccharide activated the NF‐κB signaling pathway, which upregulated HMGCR expression and promoted cholesterol accumulation in liver cancer cells [59]. In the present study, we found that upregulated HMGCR expression coexist with cholesterol accumulation in senescent FLSs, suggesting the feedback regulation of HMGCR by cholesterol was impaired in this pathological condition. Based on the data on the AKT1–Insig1 interaction, it is suggested that AKT1 activation through phosphorylation at Ser473 could be an important mechanism for the disruption of this feedback regulation of HMGCR.

However, this study has several limitations. First, the signal of AKT1 activation in senescent cells is still unclear and needs to be studied in the future studies. Second, this study mainly focused on FLSs, but the roles and mechanisms of HMGCR‐driven cholesterol metabolism in other key cell types such as chondrocytes and synovial macrophages in OA still need to be further studied. Third, the experiments in aged animals should be performed to better reflect elderly OA patients. Moreover, experiments involving the isolation and functional validation of primary HMGCR^+^ synovial fibroblasts need to be further performed. As HMGCR protein is located in the cytoplasm, it is technically challenging to obtain viable HMGCR^+^ primary synovial fibroblasts from wild‐type mice by flow cytometric sorting. In future studies, we will employ genetic engineering strategies to generate HMGCR fluorescent reporter mice, thereby providing technical support for the isolation of viable HMGCR^+^ primary synovial fibroblasts.

## 4 Conclusion

This study found an increase of HMGCR^+^ FLSs within the OA synovium exhibiting senescence phenotype, promoting OA progression. Synovial FLS‐targeted inhibition of HMGCR effectively alleviated cellular senescence of FLSs, pain‐related behaviors, synovitis, and cartilage degradation in a DMM‐induced OA mouse model. Mechanistically, phosphorylation of AKT1 at Ser473 enhanced its binding to Lys140 of Insig1, which facilitated the formation of the AKT1–Insig1 complex. Subsequently, activated AKT1 promoted phosphorylation of Insig1 at Ser189, potentially enhancing the dissociation of Insig1 from SCAP, thereby contributing to increased HMGCR transcription and increased cellular senescence in FLSs (Figure 9). Our study reveals a novel mechanism of HMGCR‐driven cellular senescence involving disruption of cholesterol negative‐feedback regulation by AKT activation in the pathological context of OA, which opens a new avenue for disease‐modifying OA therapy in the future. Moreover, our mechanistic insights may provide a valuable reference for understanding the pathogenesis of other age‐related diseases characterized by cholesterol dysregulation and cellular senescence.

**FIGURE 9:**
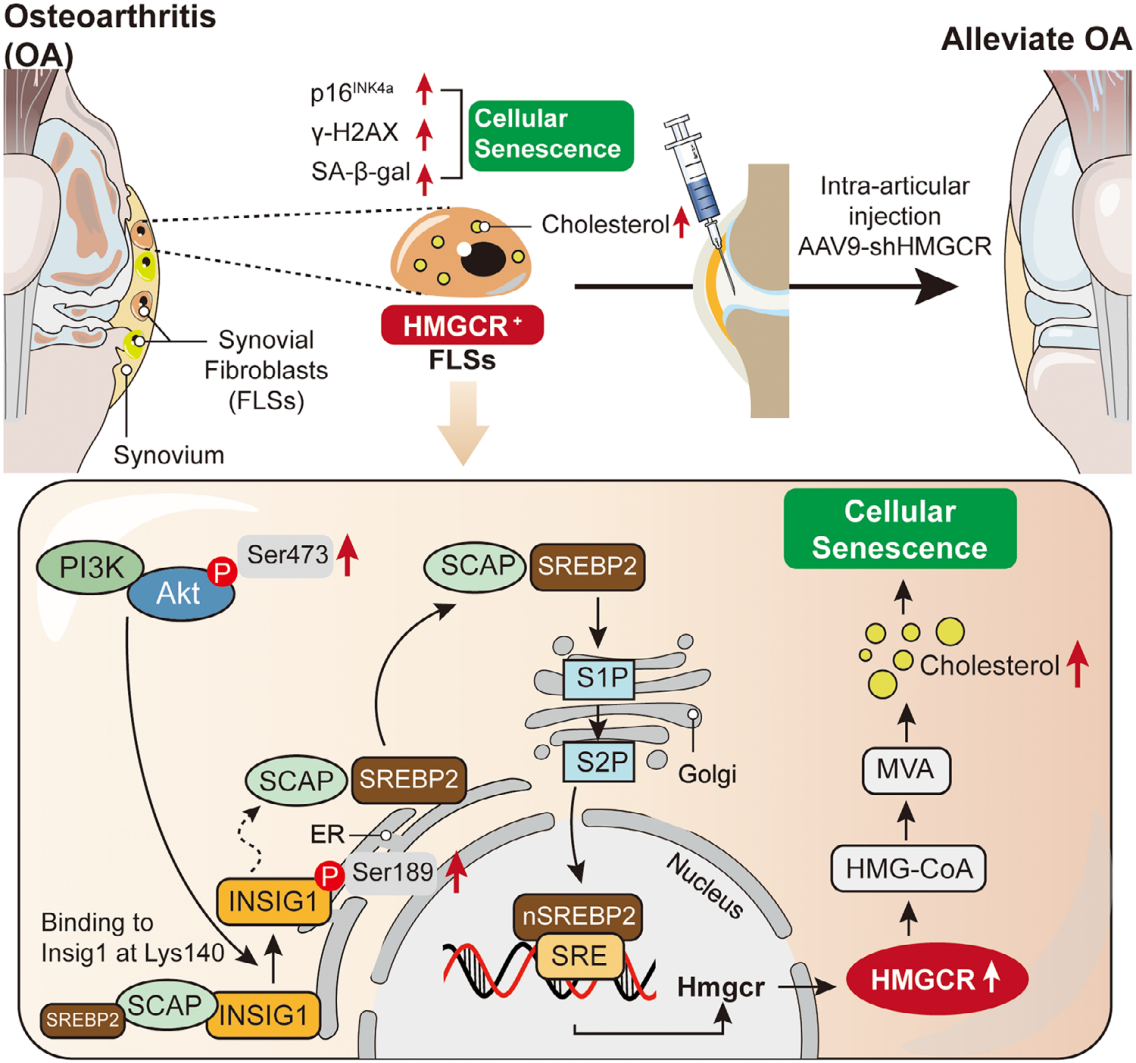
Working model.

## 5 Experimental Methods

### 5.1 Human Synovial Tissues and Primary Fibroblasts from OA Patients

Human OA synovial tissues were obtained from 6 patients undergoing total knee replacement at the Daping Hospital of Chongqing. The control synovial tissues were collected from 6 patients who underwent meniscoplasty with mild cartilage damage or synovitis. Detailed patient characteristics are presented in Table S7. Written informed consents were obtained from all recruited patients. This clinical study was approved by the Ethics Committee of the PLA Army Characteristic Medical Center for medical investigation (Approval No: 2022‐235).

Primary fibroblasts were isolated from OA patients undergoing total knee replacement (OA‐FLSs). Synovial tissues obtained from the operating room were placed on a clean bench where adjacent adipose, ligaments, and bone tissues were removed. These tissues were then digested with 0.25% trypsinase (Gibco/Life Technologies, USA) at 37°C for 15 min. Synovial tissues were cut into small pieces. Finally, primary fibroblasts were isolated from these synovial tissues by additional digestion with 0.1% collagenase I (Gibco/Life Technologies, USA) overnight at 37°C in a 5% CO_2_ incubator. Cells were seeded in 35 mm diameter dishes, cultured in human synovial fibroblast complete culture medium (Procell) and incubated under the same conditions. The culture medium was changed once every other day.

### 5.2 Cell Culture and Cellular Senescence Model

NIH‐3T3 cells (Procell) were cultured in Dulbecco's Modified Eagle Medium (DMEM; Thermo Fisher Scientific), supplemented with 10% Newborn Calf Serum (NCS, Thermo Fisher Scientific) and 1% penicillin/streptomycin (P/S; HyClone) at 37°C in a humidified atmosphere containing 5% CO_2_.

Induction of senescence by double treatment of TNF‐α was modified from the published methodologies [60, 61, 62]. In brief, NIH‐3T3 cells were cultured in complete DMEM with 10 ng mL^−1^ TNF‐α (R&D, 410‐MT) for 24 h. After washing, cells were cultured in TNF‐α‐free DMEM for 12 h, followed by stimulation with 20 ng mL^−1^ TNF‐α for 8 h.

Simvastatin (Sim; MCE) was activated by sodium hydroxide (NaOH) before use. Briefly, 2 mg Sim was dissolved in 50 µL anhydrous ethanol. Then, 75 µL 0.1 n NaOH was added to the solution. The mixture was then incubated at 50°C for 2 h. Thereafter, the solution was neutralized to pH 7.2 with HCl and brought to a final volume of 250 µL with deionized water.

### 5.3 Plasmids and Transfection

The pDNA3.1‐U6‐shRNA‐CMV‐EGFP plasmid was designed to co‐express HMGCR‐targeting shRNAs and EGFP. Three shRNAs against murine HMGCR were engineered: shRNA1: 5′‐GAGTTCAAACTGTATTACTTT‐3′; shRNA2: 5′‐GCATGTCAGTGTTGGCCAACT‐3′; shRNA3: 5′‐AACTGTATTACTTTAATGGAA‐3′. The other three shRNAs against human HMGCR were engineered: shRNA1: 5′‐GATCGCTATGATTGAGGTCAACATTACTCGAGTAATGTTGACCTCAATCATAGTTTTTT‐3′; shRNA2: 5′‐ GATCGGCTTGTGTGTCCTTGGTATTACTCGAGTAATACCAAGGACACACAAGCTTTTTT‐3′; shRNA3: 5′‐ GATCGGCCGACAGTTACTTTCCAAGACTCGAGTCTTGGAAAGTAACTGTCGGCTTTTTT‐3′. Each shRNA sequence was cloned downstream of the U6 promoter, with the CMV promoter driving EGFP expression for transfection monitoring. Plasmid construction was performed by Tsingke Biotechnology Co., Ltd. (Beijing, China).

PCR‐amplified mouse wild‐type and mutant Insig1 were cloned into pcDNA3.1(+)‐3xFlag. The wild‐type and mutant AKT1 were cloned into pcDNA3.1(+)‐3xMyc. These expression plasmids were purchased from Public Protein/Plasmid Library (Nanjing, China).

NIH‐3T3 cells or OA‐FLSs were seeded in 6‐well or 12‐well plates. At 80% confluency, transfections were performed using Lipofectamine 3000 (Thermo Fisher Scientific) according to the manufacturer's protocol.

### 5.4 Animal Model

The OA mouse model was induced by DMM surgery on the right knee of 12‐week‐old C57BL/6 mice [63]. In brief, mice was anesthetized and the right knee underwent aseptic preparation for surgery. The joint capsule medial to the patellar tendon was incised. Following exposure of the intercondylar region, the meniscotibial ligament was transected. The joint capsule and skin were then sutured. In sham‐operated controls, the right joint capsule was incised but the meniscotibial ligament remained intact. All animal experimental procedures were approved by the Laboratory Animal Welfare and Ethics Committee of the Army Medical University (Approval No: AMUWEC20237408).

### 5.5 AAV Production and Intra‐Articular Administration

As previously reported [23, 24], a recombinant adeno‐associated virus serotype 9 (AAV9) engineered with the synoviocyte‐targeting peptide HAP‐1 (designated AAV9.HAP‐1) was constructed to deliver HMGCR‐targeting shRNA in the murine synovium. Meanwhile, the Fap promoter and shRNA2 were selected as the promoter and HMGCR‐targeting shRNA to further enhance the targeting efficiency of AAV to the synovial membrane in mouse joints. AAV9.HAP‐1‐shHMGCR (AAV‐shHMGCR), AAV9.HAP‐1‐scramble (AAV‐Ctrl), and AAV9.HAP‐1 (AAV‐Vector) were commercially synthesized by Tsingke company (Beijing, China). For local delivery of HMGCR‐targeting shRNA, 10 µL AAV was administered to mice via intra‐articular injection at the second and fifth weeks after DMM surgery. All mice were euthanized at 8 weeks post‐surgery.

### 5.6 WB

Protein extracts from dTNF‐InSen NIH‐3T3 cells were prepared using RIPA lysis buffer (Beyotime) with protease inhibitors (Roche). After quantification, 20 µg samples were separated by 12% sodium dodecyl sulfate–polyacrylamide gel electrophoresis (SDS‐PAGE) and transferred to PVDF membranes (Millipore). After blocking the membrane for 1 h using 5% nonfat milk dissolved in PBST (1×PBS + 0.5% Tween‐20), specific primary antibodies were added and incubated overnight at 4°C. After three PBST washes, the membranes were incubated with species‐matched secondary antibodies at 37°C for 1 h. The membranes were washed three times with PBST before detecting immunoreactivity via chemiluminescence. Finally, chemiluminescent signals were visualized using the ChemiScope 6000 system (Clinx Science Instruments). The primary antibodies were as follows: HMGCR (Huabo, ET1702‐41), p16 (Abcam, ab189034), p53 (CST, 2524S), p21 (CST, 37543T), AKT1 (CST, 2938S), p‐AKT^ser473^ (ABclonal, AP0637), Insig1 (Santa, sc‐390504), phosphoserine/threonine (p‐Ser/Thr; BD Biosciences, 612549), SCAP (Proteintech, 12266‐1‐AP), IL‐6 (Proteintech, 26404‐1‐AP), MMP13 (Abcam, ab39012), Myc (CST, 2276), Flag (CST, 14793), β‐actin (Proteintech, 66009‐1‐AP).

### 5.7 Co‐IP

NIH‐3T3 cells were lysed with 1 mL RIPA containing protease inhibitor. The protein‐containing supernatant was collected, of which 100 µL lysate was mixed with SDS‐PAGE Loading Buffer for WB analysis. The remaining 900 µL lysate was reserved for Co‐IP. Subsequently, 400 µL wash buffer was added to 30 µL protein A/G magnetic beads (MCE), thoroughly resuspended, and placed on a magnetic stand for 1 min. After magnetic separation, the supernatant was discarded. This washing procedure was repeated twice. 900 µL of cell lysate was combined with 30 µL magnetic beads and 10 µL antibody. The mixture was thoroughly resuspended and incubated overnight at 4°C on a rotary mixer. Then, magnetic separation was performed and the supernatant was discarded. The beads were then washed four times with 400 µL wash buffer. Subsequently, 30 µL SDS‐PAGE Loading Buffer was added to the washed beads, mixed thoroughly, and heated at 100°C for 10 min in a heating block. After magnetic separation, the eluted supernatant was collected for SDS‐PAGE analysis.

### 5.8 Quantitative Real‑Time PCR Assay (RT‐qPCR)

Using TRIzol reagent (Invitrogen), total RNA was extracted from NIH‐3T3 cells. The isolated RNA was reverse transcribed into cDNA for qPCR analysis. qPCR reactions were run on an Mx3000P system (Takara) with the SYBR Green RT‐PCR Kit (Takara). Threshold cycle (*C*_t_) values were recorded, and relative mRNA expression levels were calculated via the ΔΔ*C*_t_ method with mouse Gapdh mRNA as the endogenous control. All qRT‐PCR assays are conducted in triplicate with gene‐specific primer pairs that are listed in Table S8.

### 5.9 Sample Preparation

Mouse knee joints were fixed with 4% paraformaldehyde (PFA) at 4°C for 48 h, followed by decalcification for 14 days in 0.5 m ethylenediaminetetraacetic acid (EDTA) chelating solution (Solarbio) at 4°C. Human synovial tissues were fixed only with 4% PFA. For paraffin sections, samples were dehydrated in ethanol, embedded in paraffin and cut into 5 µm sections. For frozen sections, samples were infiltrated with 30% sucrose, embedded in Optimal Cutting Temperature (OCT; Thermo Fisher Scientific) compound, and sectioned into 10 µm thick cryosections.

### 5.10 Histological Analysis

Paraffin‐embedded tissue specimens were first dewaxed in xylene and then rehydrated through a graded ethanol series. For immunohistochemical staining, knee joint sections underwent trypsin‐based antigen retrieval and were subsequently blocked with goat serum. After incubation with the primary antibody overnight, the secondary antibody and horseradish peroxidase‐labeled streptavidin biotin were added. Sections were counterstained with methyl green or hematoxylin. The primary antibody was: HMGCR (Huabo, ET1702‐41).

Safranin O/fast green staining was used to assess cartilage injury based on the OARSI scoring system [64]. Knee joints from mice were scored from 0 to 6 according to histologic changes. H&E staining revealed synovial inflammation, which was scored using reported methods [64]. Briefly, the scoring system ranges from 0 to 9 based on histological changes in the mouse synovium. The overall score comprises three individually evaluated metrics: the degree of synovial intima hyperplasia (0–3 points), the extent of inflammation in synovial subintima (0–3 points), and the presence of angiogenesis (0–3 points).

### 5.11 Immunofluorescent Staining

Cells were fixed with 4% paraformaldehyde for 10 min. Tissue cryosections were gently rehydrated in PBS for 3–5 min. Subsequently, tissue sections were blocked with immunostaining blocking buffer (Beyotime) at 37°C for 1 h, followed by incubation with primary antibodies overnight at 4°C. After washing, secondary antibodies (Thermo Fisher Scientific) were applied to the samples for 1 h at room temperature. Nuclei were counterstained using DAPI and fluorescence images were acquired using a fluorescence microscope (Olympus). The primary antibodies were as follows: HMGCR (Invitrogen, PA5‐116675), HMGCR (Santa, sc‐271595), p16 (Abcam, ab189034), PDGFR‐α (R&D, AF1063), γ‐H2AX (CST, 9718S), p53 (CST, 2524S).

### 5.12 LC–MS/MS Untargeted Metabolomics Analysis

Sample preparation and metabolomic profiling were performed by Shanghai Applied Protein Technology Co., Ltd., according to standard operating procedures. Samples were separated using a Vanquish LC ultra‐high‐performance liquid chromatography (Vanquish UHPLC, Thermo) system and analyzed using an Orbitrap Exploris 480 mass spectrometer (Thermo). Mass spectrometry detection was performed in both positive and negative electrospray ionization (ESI) modes.

For HILIC separation, samples were analyzed using a 2.1 mm × 100 mm ACQUITY UPLC BEH Amide 1.7 µm column (Waters, Ireland). In both ESI positive and negative modes, the mobile phase contained A = 25 mm ammonium acetate and 25 mm ammonium hydroxide in water and B = acetonitrile. The gradient was 95% B for 0.5 min and was linearly reduced to 65% in 6.5 min, then reduced to 40% in 1 min and maintained for 1 min, then increased to 95% in 0.1 min and kept for 2.9 min.

The ESI source conditions were set as follows: Ion Source Gas1 (Gas1) as 50, Ion Source Gas2 (Gas2) as 2, source temperature: 350°C, IonSpray Voltage Floating (ISVF): +3500 V/−2800 V. In MS‐only acquisition, the instrument was set to acquire over the m/z range 70–1200 Da, the resolution was set at 60 000 and the accumulation time was set at 100 ms. In auto MS/MS acquisition, the instrument was set to acquire over the m/z range 70–1200 Da, the resolution was set at 60 000 and the accumulation time was set at 100 ms with an exclusion time of 4 s.

Data analysis, including univariate and multivariate statistical analysis, screening for differential metabolites, and bioinformatic analysis, was conducted using the APT‐BioCloud platform. VIP > 1 and *p* value < 0.05 were used to screen for significantly changed metabolites.

### 5.13 SA‐β‐gal Staining

SA‐β‐gal staining (Beyotime) was performed to evaluate β‐galactosidase activity, according to the manufacturer's guidelines. Briefly, cells were fixed at room temperature for 15 min followed by PBS washes, prior to overnight incubation with SA‐β‐gal staining solution at 37°C. A minimum of three random microscopic fields per experimental group were imaged, and SA‐β‐gal^+^ cells were counted using ImageJ.

### 5.14 Cell Cycle Measurement

Propidium iodide (PI) staining was used for cell cycle analysis according to the instructions of the cell cycle detection kit (Vazyme). The cells were detached with 0.25% trypsinase to obtain cell pellets. 1 mL of PI staining solution was added to the cell pellets, followed by the addition of 5 µL of cell membrane permeabilization reagent. The mixture was gently pipetted to ensure thorough mixing. After cell resuspension, the sample was incubated in the dark at 37°C for 1 h. Red fluorescence was detected using flow cytometry at an excitation wavelength of 488 nm, and light scattering was simultaneously measured. Flowjo 10.10 software was used for DNA content analysis and cell cycle determination.

### 5.15 Serum Measurement

Blood plasma was collected from mouse heart. The sample was centrifuged at 4°C and 1500× *g* for 15 min. The supernatant was collected and used as the serum sample for analysis. Serum parameters including total cholesterol (TC, Elabscience, E‐BC‐K109‐M) and low‐density lipoprotein cholesterol (LDL, Elabscience, E‐BC‐K205‐M) were measured using commercial kits according to the manufacturers’ manuals.

### 5.16 Nile Red

NIH‐3T3 cells grown on glass coverslips were fixed with 4% PFA (Solarbio) for 30 min at room temperature, followed by PBS washes. Subsequently, NIH‐3T3 cells were incubated with 1 µm Nile Red working solution (Solarbio) for 15 min at room temperature. Then, NIH‐3T3 cells were washed twice with PBS and counterstained with DAPI for 10 min. Confocal images were acquired using a fluorescence microscope (Olympus).

### 5.17 Gait Analysis

Mouse gait parameters were quantitatively assessed at 8 weeks after DMM surgery using the CatWalk XT system (Noldus Information Technology). All mice underwent 2 days of runway acclimatization until achieving consistent velocity profiles without hesitation prior to initial assessment. Paw prints were visualized via LED light refraction through the glass platform and captured by video imaging (CatWalk XT). A minimum of three valid step cycles per mouse were quantified per time point using CatWalk software v10.6 (Noldus). The Duty Cycle was computed: Duty Cycle (%) = Stand / (Stand + Swing) × 100%.

### 5.18 Open Field Test

The open‐field apparatus, a chamber measuring 50 × 50 × 45 cm, was constructed with white PVC walls and a grid beam system. Mice were gently positioned in the chamber's center. Behavioral data were acquired using Noldus Ethovision XT 12.1 software (Noldus) [65]. Locomotor activity was quantified by the total distance traveled for 5 min per mouse.

### 5.19 Weight Bearing Test

Spontaneous pain and postural equilibrium were assessed using the static weight‐bearing test (Bioseb) [66]. Mice were minimally restrained and positioned comfortably in the apparatus holder, allowing their hind paws to rest independently on dual sensor plates. The natural weight distribution between hindlimbs, which reflects discomfort in an injured paw, was measured. Once the animal acclimated, weight distribution was recorded for 10 s and then averaged. To quantify asymmetry and pain‐related behavior, the weight distribution ratio (Right/Left hindlimb) was calculated.

### 5.20 ChIP‐qPCR Assay

ChIP‐qPCR primers targeting HMGCR (containing SRE site) and HMGCR (negative control) were designed using sequences from Table S9 for subsequent experiments. ChIP experiments were performed using ChIP Assay Kit (Beyotime, P2078) according to the manufacturer's instructions. Briefly, the dTNF‐InSen FLSs were cross‐linked with 1% formaldehyde for 10 min at room temperature. The cell pellet was resuspended in 0.2 mL of SDS lysis buffer supplemented with 1 mm phenylmethylsulfonyl fluoride (PMSF) and incubated on ice for 10 min. Sonication (30 s pulse and 30 s pause, 3 cycles) was used for complete lysis of nuclei in ChIP buffer containing 1 mm PMSF. For immunoprecipitation, digested chromatin was incubated with 5 µg of SREBP2 antibody overnight at 4°C with rotation. After that, magnetic beads were added to the immunoprecipitation reaction for 2 h at 4°C with rotation. According to the manufacturer's instructions, immunoprecipitated chromatin DNA was eluted and quantified using real‐time quantitative PCR. The primary antibodies were as follows: SREBP2 (R&D, AF7119), Goat IgG (GeneTex, GTX35039).

### 5.21 HMGCR‐Luc Construction and Luciferase Reporter Assay

The mouse HMGCR promoter region (−2.0 kb to +100 bp relative to the transcription start site) was inserted into the pGL3‐Basic firefly luciferase vector to produce the HMGCR(WT)‐Luc plasmid. This construct was custom‐synthesized by Tsingke. Based on prior research [39, 67], two SRE‐mutant luciferase reporters were generated via site‐directed mutagenesis: HMGCR‐Luc (SRE1‐Mut) (mutating GTGGTGAGAG to GaaaattttG); HMGCR‐Luc (SRE2‐Mut) (ATGGTGCGGTGCC mutated to ATaaaaattTttC). Subsequently, the above luciferase reporters into NIH‐3T3 cells via Lipofectamine 3000 were transfected and then induced cellular senescence using TNF‐α. Luciferase activity was analyzed by Dual‐Luciferase Reporter Assay System (Promega), normalized to co‐transfected Renilla luciferase (pRL‐TK). These mutant plasmids were custom‐synthesized by the Public Protein/Plasmid Library (Nanjing, China).

### 5.22 scRNA‐seq

scRNA‐seq data (GSE211584) were obtained from the Gene Expression Omnibus (NCBI GEO) repository [20]. Synovial cells were reclassified into 7 clusters using marker genes from the CellMarker 2.0 public database [68]. FLSs expressed Pdgfra and Fap; macrophages were characterized by expression of Adgre1; endothelial cells expressed Pecam1 and Cdh5; tenocytes expressed Scx and Tnmd; Schwann cells expressed Sox10; pericytes expressed Rgs5 and Myh11; lymphoendothelial cells expressed Lyve1. Subsequently, GO enrichment analysis and GSEA in HMGCR^+^ and HMGCR^−^ FLSs were performed. Further validation confirmed significant enrichment of senescence‐associated genes in HMGCR^+^ FLSs.

### 5.23 Molecular Docking and Dynamics

Protein–protein docking was performed between mouse Insig1 and AKT1 or pAKT1 (Ser473+Thr308) using HDOCK 2.3 software. Structures were obtained from AlphaFold databases, prepared by removing nonprotein entities and assigning charges. Subsequently, MD simulations were performed using GROMACS 2022 based on the results of the molecular docking. The complex was solvated in a TIP3P water box and neutralized with counterions. The AMBER99SB‐ILDN force field was used for the protein. The system underwent 50 000‐step energy minimization via the steepest descent algorithm, followed by 100 ps NVT (constant number, volume, and temperature) and 100 ps NPT (constant number, pressure, and temperature) equilibration with position restraints on protein heavy atoms. A 100 ns unrestrained production simulation was then conducted at 310 K and 1 bar, using the V‐rescale thermostat and Parrinello–Rahman barostat. The calculations were performed by Weihuan Biological Technology Co., Ltd. (Shanghai, China).

### 5.24 Statistical Analysis

All statistics were computed in SPSS (version 27.0.1). Data normality was assessed using the Shapiro–Wilk test. For non‐normally distributed data comparing two unpaired groups, the Mann–Whitney *U* test was applied. Normally distributed data were analyzed using Student's *t*‐test (equal variances) or Welch's *t*‐test (unequal variances). Normally distributed data with homogeneous variances underwent one‐way ANOVA with Bonferroni post hoc testing. For normally distributed data exhibiting heterogeneous variances, Tamhane's T2 post hoc test was applied. Non‐normally distributed data were analyzed using the Kruskal–Wallis test. For categorical data, the Pearson chi‐square test was used for group comparisons; Fisher's exact test was applied when the total sample size was less than 40 or when the expected frequency in any cell was less than 5. Statistical significance was defined as *p* < 0.05. Data in the graphs were presented as the mean ± standard error of the mean. Sample size (*n*) for each statistical analysis are presented in figure legends. To control false discovery rates, *p*‐values underwent multiplicity correction using Bonferroni, Tamhane's T2, or Benjamini–Hochberg procedures.

## Author Contributions

L.C., B.Z., and Z.N. were involved in the conception and design of the research. X.Z., X.L., and B.Z. assumed primary responsibility for performing the majority of the experiments. X.L. and Y.T. performed the analysis of relevant data. X.P., S.L., and B.L. contributed to the establishment of the DMM mouse model. H.W., S.F., B.H., and B.L. assisted with the histological staining. R.J. was responsible for collecting synovial tissues from clinical patients and preparing histological sections. C.B., W.Z., and L.C. provided daily care for some experimental animals. S.Z. designed the schematic diagrams and assembled the figures. X.Z., S.Z., and X.L. wrote the manuscript. L.C., B.Z., and Z.N. revised the manuscript. All authors reviewed and approved the final version for publication.

## Ethics Statement

The animal experiments in the current study were approved by the Laboratory Animal Welfare and Ethics Committee of the Army Medical University (Approval No: AMUWEC20237408). Collection of clinical samples was approved by the Ethics Committee of the PLA Army Characteristic Medical Center (Approval No: 2022‐235).

## Conflicts of Interest

The authors declare no conflicts of interest.

## Supporting information




**Supporting File 1**: advs76498‐sup‐0001‐SuppMat.docx.

**Supporting File 2**: advs76498‐sup‐0002‐TableS1‐S9.zip.

## Data Availability

The data that support the findings of this study are available from the corresponding author upon reasonable request.
