# Supplementary material for: HMGCR‐Driven Cholesterol Metabolism Promotes Osteoarthritis Progression by Accelerating Synovial Fibroblast Senescence
Source: Adv Sci (Weinh). 2026 Jul 13;13(55):e76498. doi: 10.1002/advs.76498 (PMC13360124; doi:10.1002/advs.76498)
Supplement: Supplementary file 1 [file ADVS-13-e76498-s001.docx]

**Figure S1.** (**A** and **B**) Spearman’s correlation analysis between percentages of HMGCR⁺ PDGFR-α^+^ cells and clinical indicators, including Kellgren-Lawrence grade and pain score. n = 12 each group. All statistical tests were two-sided.

**Figure S2.** (**A** and **B**) Quantitative results for WB analysis in **Fig. 3B** and **Fig. 3F**. n = 3 each group. (**C** and **D**) Certain shRNAs were used to knockdown the protein level of HMGCR in the dTNF-InSen NIH-3T3 cells and primary human OA synovial fibroblasts (OA-FLSs). (**E**) Representative immunofluorescence staining and quantification of p16^+^ cells among HMGCR^-^ and HMGCR^+^ OA-FLSs. n = 3 each group. All statistical tests were two-sided. Student's *t*-test (**A**, **E**). One-way ANOVA with Bonferroni test (**B**). dTNF-InSen, induction of senescence by double treatment of TNF-α.

**Figure S3.** (**A** and **B**) Representative images of Nile red staining and quantitation of mean Nile red fluorescence intensity in synovial tissues from OA patients and DMM-induced OA mice, compared with the normal control. n = 3 per group. (**C** and **D**) Typical staining images of Nile red and quantitation of mean Nile red fluorescence intensity following treatment with 0.5 µM Sim or transfection with shHMGCR plasmid in dTNF-InSen fibroblasts. n =3 each group. All statistical tests were two-sided. Student's *t*-test (**A**, **B**). One-way ANOVA with Bonferroni test (**C, D**). Sim, simvastatin; MFI, Mean Fluorescence Intensity.

**Figure S4.** Quantitative results for WB analysis in **Fig. 5D**. n = 3 each group. All statistical tests were two-sided. One-way ANOVA with Bonferroni test.

**Figure S5.** **Protein-protein docking and molecular dynamics simulation between AKT1 and Insig1.** (**A**) AKT1-Insig1 complex surface in protein-protein docking. (**B**) The molecular dynamics simulations showing the interaction between AKT1 and Insig1 ranging from 0 to 100ns. (**C**) Rg showing that the AKT1-Insig1 complex remained in a stable state throughout the MD simulation process from 0 to 100ns. (**D**) The molecular dynamics simulations of p-AKT1-Insig1 complex ranging from 0 to 100ns. (**E** and **F**) RMSD and RMSF showing the binding of p-AKT1 and Insig1. Red, black and blue color represented p-AKT1, Insig1 and the complex respectively. The black arrow represents the phosphorylation site. (**G**) Rg of p-AKT1-Insig1 complex throughout the MD simulation process from 0 to 100ns. (**H** and **I**) Hydrogen bonding and hydrophobic interactions in the AKT1-Insig1 and p-AKT1-Insig1 complexes. The section marked by the dashed line has been shown in **Fig. 6E**. Rg, radius of gyration; MD, molecular dynamics; RMSD, protein backbone root mean square deviation; RMSF, root mean square fluctuation.

**Figure S6.** (**A**-**D**) Schematic representation of wild-type and point-mutated plasmid constructs used in **Fig. 6**. (**E**) mRNA expression of HMGCR in the dTNF-InSen FLSs after transfection of Insig1 K140A or Insig1 WT vector under the condition of AKT1 WT overexpression. n = 3 each group. (**F**) Schematic representation of the point-mutated plasmid constructs used in **Fig. 7**. All statistical tests were two-sided. Student's *t*-test (**E**).

**Figure S7.** (**A** and **B**) Serum LDL-cholesterol and total cholesterol levels in experimental mice. n = 6 per group. (**C**) Representative images of EGFP fluorescence using the animal live imaging equipment. (**D** and **E**) EGFP fluorescence intensity and H&E staining in individual organs and tissues (heart, liver, spleen, lung, kidney, and keen joint) from mice after intra-articular injection of AAV-Ctrl or AAV-shHMGCR. (**F**) Static weight bearing test assessing hindlimb weight distribution (RH/LH) in mice after different treatment. n = 5 per group. (**G**) Open field test evaluating the travel distance during a 5-min session in the chamber. n = 5 per group. (**H**) Representative images of Safranin O/fast green and H&E staining across the four groups (**left**). The extent of synovial inflammation quantified by the synovitis score and the severity of cartilage damage quantified by the OARSI scoring system among the four groups (**right**). n = 5 for each group. The intra-articular injection of 10 μL AAV (1×10¹¹ GC) was administered at the second and fifth weeks after DMM surgery. Behavioral tests and pathological examination were conducted at 8 weeks after DMM. All statistical tests were two-sided. One-way ANOVA with Bonferroni test (**A**, **B**, **F**, and **G**). Kruskal-Wallis test (**H**). RH, right hindlimb; LH, left hindlimb.
